# Design and testing of a humanized porcine donor for xenotransplantation

**DOI:** 10.1038/s41586-023-06594-4

**Published:** 2023-10-11

**Authors:** Ranjith P. Anand, Jacob V. Layer, David Heja, Takayuki Hirose, Grace Lassiter, Daniel J. Firl, Violette B. Paragas, Adam Akkad, Sagar Chhangawala, Robert B. Colvin, Russell J. Ernst, Nicholas Esch, Kristen Getchell, Alexandra K. Griffin, Xiaoyun Guo, Katherine C. Hall, Paula Hamilton, Lokesh A. Kalekar, Yinan Kan, Ahmad Karadagi, Feng Li, Susan C. Low, Rudy Matheson, Claudia Nehring, Ryo Otsuka, Matthew Pandelakis, Robert A. Policastro, Rebecca Pols, Luis Queiroz, Ivy A. Rosales, William T. Serkin, Kathryn Stiede, Toshihide Tomosugi, Yongqiang Xue, Gabriel E. Zentner, David Angeles-Albores, J. Chris Chao, Juliet N. Crabtree, Sierra Harken, Nicole Hinkle, Tania Lemos, Mailin Li, Lorena Pantano, Denise Stevens, Omar D. Subedar, Xiaoqing Tan, Shiyi Yin, Imran J. Anwar, David Aufhauser, Saverio Capuano, Dixon B. Kaufman, Stuart J. Knechtle, Jean Kwun, Dhanansayan Shanmuganayagam, James F. Markmann, George M. Church, Mike Curtis, Tatsuo Kawai, Michele E. Youd, Wenning Qin

**Affiliations:** 1 eGenesis, Cambridge, MA USA; 2 Center for Transplantation Sciences, Massachusetts General Hospital, Harvard Medical School, Boston, MA USA; 3 Department of Pathology, Massachusetts General Hospital, Harvard Medical School, Boston, MA USA; 4 Duke Transplant Center, Department of Surgery, Duke University Medical Center, Durham, NC USA; 5 Department of Surgery, Division of Transplantation, School of Medicine and Public Health, University of Wisconsin, Madison, WI USA; 6 Wisconsin National Primate Research Center, Madison, WI USA; 7 Department of Animal and Dairy Science, University of Wisconsin, Madison, WI USA; 8 Department of Genetics, Harvard Medical School, Boston, MA USA; 9 Wyss Institute of Biologically Inspired Engineering, Harvard University, Cambridge, MA USA

**Keywords:** Synthetic biology, Preclinical research

## Abstract

Recent human decedent model studies^1,2^ and compassionate xenograft use^3^ have explored the promise of porcine organs for human transplantation. To proceed to human studies, a clinically ready porcine donor must be engineered and its xenograft successfully tested in nonhuman primates. Here we describe the design, creation and long-term life-supporting function of kidney grafts from a genetically engineered porcine donor transplanted into a cynomolgus monkey model. The porcine donor was engineered to carry 69 genomic edits, eliminating glycan antigens, overexpressing human transgenes and inactivating porcine endogenous retroviruses. In vitro functional analyses showed that the edited kidney endothelial cells modulated inflammation to an extent that was indistinguishable from that of human endothelial cells, suggesting that these edited cells acquired a high level of human immune compatibility. When transplanted into cynomolgus monkeys, the kidneys with three glycan antigen knockouts alone experienced poor graft survival, whereas those with glycan antigen knockouts and human transgene expression demonstrated significantly longer survival time, suggesting the benefit of human transgene expression in vivo. These results show that preclinical studies of renal xenotransplantation could be successfully conducted in nonhuman primates and bring us closer to clinical trials of genetically engineered porcine renal grafts.

## Abstract (web summary)

Using kidneys from a genetically engineered porcine donor transplanted into a cynomolgus monkey model, the design, creation and long-term function of kidney grafts supporting life are explored.

## Main

Xenotransplantation may offer a transformative solution to the worldwide organ shortage crisis^1–3^. To proceed to clinical studies, a clinically ready porcine donor must be engineered and its xenograft successfully tested in a nonhuman primate (NHP) model to assess its safety and efficacy.

Over the years, various genetically engineered porcine donors have been created and their kidneys transplanted into Old World monkeys (OWMs)^4–6^. Although these donors contributed to our understanding of molecular incompatibilities in xenotransplantation, they are not clinically ready. First, the donors were often created on a commercial pig breed whose heart and kidney sizes are too large for human application. Although elimination of growth hormone receptor gene expression could reduce organ sizes^2,3^, it comes with other undesired biological consequences^7^. Second, the donors were designed for testing in OWMs. They lacked the α-Gal (galactose-α-1,3-galactose) or the α-Gal and Sd(a) (Sia-α2.3-[GalNAc-β1.4]Gal-β1.4-GlcNAc) glycans but expressed the Neu5Gc (*N*-glycolylneuraminic acid) glycan to match with Neu5Gc expression in OWMs. However, in vitro analysis suggests that a human-compatible porcine donor should ideally have all three glycans eliminated to match with the absence of the three glycans in humans^8,9^. Although renal grafts derived from the porcine donors lacking these three glycans and carrying various human transgenes have been tested in OWMs, graft survival was short^8^ or not all human transgenes were expressed^10^. Third, the donors carried porcine endogenous retrovirus (PERV) sequences in their genome, which present a zoonotic risk, as PERV transmission to human cells in culture and their integration into the human genome have been demonstrated^11,12^.

Here we created a humanized porcine donor on the Yucatan miniature pig breed and transplanted porcine renal grafts lacking the three glycans with or without PERV knockout (retroviral inactivation (RI)) (referred to as 3KO.RI or 3KO), or 3KO with seven human transgenes with or without RI (referred to as 3KO.7TG.RI or 3KO.7TG, respectively) into cynomolgus monkeys (*Macaca fascicularis*). We show that a humanized porcine renal graft, combined with a clinically relevant immunosuppressive regimen, supported long-term NHP survival for up to 2 years (758 days).

## Porcine molecular incompatibilities

Substantial molecular incompatibilities exist between pigs and humans. Of particular interest to xenotransplantation are cell-surface antigens and regulators of the complement cascade, coagulation pathway, and inflammation process^13,14^ (Supplementary Table 1).

**Fig. 1:**
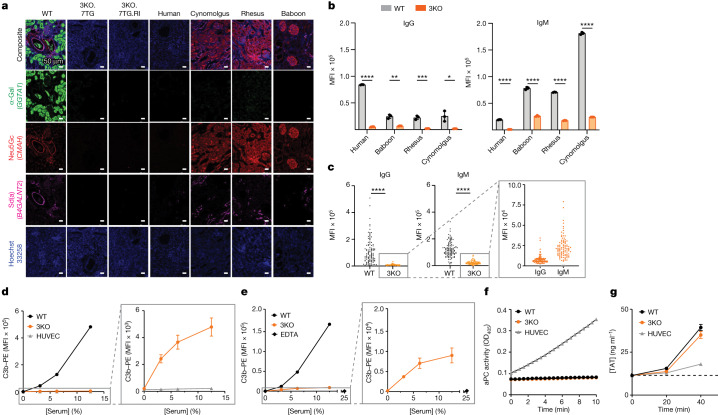
Functional incompatibilities exist between porcine donor and primate recipient. **a**, IHC confirmed that the three glycan antigens are expressed in the WT porcine kidney, but have been eliminated from the 3KO.7TG (donor ID 21077) and the 3KO.7TG.RI (donor ID A9161) kidneys, a pattern similar to that of the human kidney. In comparison, OWMs express the Neu5Gc antigen. IHC analyses of 3KO were performed for all contralateral kidney samples included in this study and 3KO phenotypes confirmed for all. **b**, WT porcine KECs bound human and NHP preformed antibodies, whereas 3KO porcine KECs showed markedly reduced antibody binding. Each sample was run with three technical replicates. Error bars are s.d. **c**, The 3KO AECs bound significantly less IgG and IgM than the WT AECs, when incubated with 96 individual cynomolgus monkey serum samples. Note that 3KO AECs retained a substantial level of IgG and IgM binding. Statistical analysis was performed using Wilcoxon matched-pairs signed rank test. **d**, WT KECs showed significant C3b deposition in human serum, compared with human umbilical vein endothelial cells (HUVECs). Although markedly reduced, the 3KO KECs retained some level of C3b deposition (right panel). Error bars are s.e.m. **e**, WT KECs showed C3b deposition and 3KO KECs showed less deposition when incubated in the cynomolgus monkey serum. Error bars are s.e.m. **f**, Porcine KECs (WT or 3KO) did not produce aPC, whereas HUVECs readily produced aPC. For **d**–**f**, WT KEC (*n* = 1), 3KO KEC (*n* = 3) and HUVEC (*n* = 1). OD_405_, optical density at 405 nm. **g**, When incubated with human whole blood, WT (*n* = 1) and 3KO (*n* = 1) porcine KECs triggered coagulation, measured as TAT formation. Error bars are s.e.m. For **d**–**g**, each point is a biological replicate examined over at least two independent experiments. With the exception of **c**, statistical analyses were performed using two-tailed unpaired Student’s *t*-tests. ****P < 0.0001, ***P < 0.001, **P < 0.01, *P < 0.05. Source Data

Porcine cells display three major glycan antigens on their cell surface — α-Gal^15^, Neu5Gc^16^ and Sd(a)^17^ (Fig. 1a) — which are the products of the corresponding glycan synthesis genes, glycoprotein α-galactosyltransferase 1 (*GGTA1*), cytidine monophospho-*N*-acetylneuraminic acid hydroxylase (*CMAH*) and β-1,4-*N*-acetyl-galactosaminyltransferase 2 (*B4GALNT2*)/B4GALNT2-like (*B4GALNT2L*). In humans, *GGTA1* (ref. ^18^) and *CMAH*^19^ evolved into pseudogenes and the α-Gal and the Neu5Gc epitopes are not expressed (Fig. 1a). After birth, humans develop antibodies to α-Gal and Neu5Gc, referred to as preformed antibodies, upon exposure to molecular mimics of these two antigens^16,20,21^. Although the human *B4GALNT2* gene is functional^22^, its expression levels vary and naturally occurring mutations have been identified that correlate with the Sd(a-) phenotype^23^. A low level of Sd(a)-reactive antibodies has been detected in humans^24^. Similar to humans, OWMs carry preformed antibodies to α-Gal and Sd(a), but unlike humans, they lack preformed antibodies to Neu5Gc, as they possess a functional *CMAH* gene^25^ (Fig. 1a).

When porcine cells are exposed to primate serum, the glycan antigens are recognized by primate preformed antibodies, leading to antibody-mediated rejection (AMR)^13^. In an antibody-binding assay, the porcine wild-type (WT) kidney endothelial cells (KECs) bound a high level of human IgG and IgM and binding was significantly reduced when the three xenoantigens were eliminated (3KO KECs) (Fig. 1b). Binding by OWM serum IgG and IgM was similar, although substantial residual antibody binding (especially IgM binding) was detected with the 3KO KECs (Fig. 1b). This is consistent with other reports that 3KO porcine cells possess additional xenoantigens recognized by OWM serum^8,9^. WT and 3KO aortic-derived endothelial cells (AECs) behaved similarly to the KECs when incubated with 96 individual cynomolgus monkey serum samples (Fig. 1c).

Antibody binding triggers complement activation, producing surface-bound C3b and soluble C3a. When incubated with human serum, human umbilical vein endothelial cells do not show C3b deposition (Fig. 1d), suggesting no antibody binding and/or complete mitigation of complement activation. When porcine WT KECs were incubated with human serum, significant C3b deposition was observed. Althoughthe 3KO KECs showed significant reduction in C3b deposition compared with the WT KECs, they retained substantial residual C3b deposition (Fig. 1d), suggesting that porcine complement regulators are less effective in mitigating human complement activation. When WT and 3KO porcine KECs were incubated with cynomolgus monkey serum, similar results were obtained, although higher residual C3b levels on 3KO porcine cells were observed, compared with those in human serum (Fig. 1e).

Under physiological conditions, thrombomodulin and endothelial protein C receptor (EPCR) are expressed on the endothelial cell surface and inhibit coagulation by enabling activated protein C (aPC)-mediated regulation^26,27^. When human thrombin and protein C were provided to human umbilical vein endothelial cells, aPC was readily generated (Fig. 1f). By contrast, aPC production was not observed when these reagents were supplied to porcine WT or 3KO KECs (Fig. 1f), suggesting that human thrombin and protein C are not compatible with porcine thrombomodulin and/or EPCR. Enhanced clotting of human whole blood ex vivo was observed, measured as thrombin–antithrombin (TAT) complex formation, probably due to the inability of the porcine cells to generate aPC (Fig. 1g).

## A humanized porcine donor

The Yucatan miniature pig breed was chosen because its organ sizes are comparable to human organs^28^. In addition, pigs with OO blood type were selected to eliminate ABO blood-type incompatibilities^29^.

**Fig. 2:**
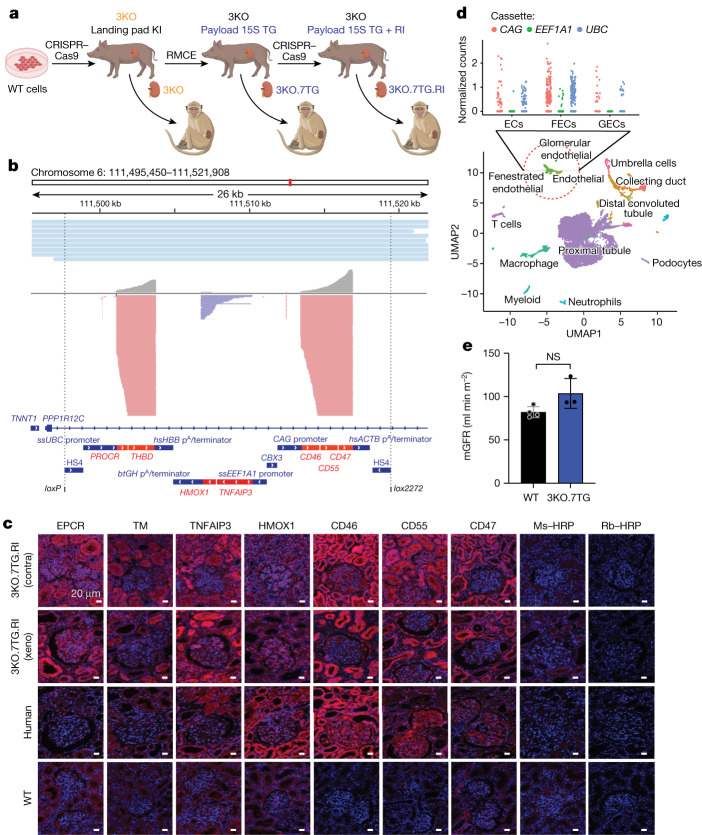
Yucatan porcine donor is engineered to carry 69 genomic edits. **a**, The porcine donor kidney, 3KO.7TG.RI, was engineered to eliminate three glycan antigens (3KO), overexpress seven human transgenes (PL15S) and inactivate PERV elements (RI) through three rounds of editing and cloning. The donor kidney, 3KO.7TG, carries 3KO and PL15S, without RI. KI, knock in; RMCE, recombinase-mediated cassette exchange. **b**, Reads from Nanopore long-read whole-genome sequencing of the 3KO.7TG.RI donor, A9161, were aligned to a custom chromosome carrying PL15S inserted at the *AAVS1* genomic safe harbour site (top). Reads from Nanopore direct RNA-seq of A9161 kidney mRNA were aligned to the custom chromosome (bottom). All three transcription units were transcribed. **c**, All seven human transgenic proteins were detected in the contralateral (contra) kidney of A9161 recovered at transplantation (row 1), and the xenograft (xeno) kidney harvested at necropsy on post-transplantation day 176 (row 2). HRP, horseradish peroxidase; TM, thrombomodulin; Ms–HRP, goat anti-mouse secondary antibody conjugated with HRP; Rb–HRP, goat anti-rabbit secondary antibody conjugated with HRP. **d**, Three endothelial cell types were identified from kidney dissociated cell populations by single-cell RNA-seq, including endothelial cells (ECs) (PECAM1^+^PLVAP^−^EHD3^−^GATA5^−^), glomerular endothelial cells (GECs) (PECAM1^+^EHD3^+^GATA5^+^) or fenestrated endothelial cells (FECs) (PECAM1^+^PLVAP^+^) (bottom), and transgene expression was examined among the three endothelial cell types (top). Mean log_2_-normalized unique molecular identifier counts were plotted against the three PL15S transcription units (*ssUBC*, *ssEEF1A1* and *CAG*). UMAP, uniform manifold approximation and projection. **e**, The 3KO.7TG porcine donors (*n* = 3) showed normal measured glomerular filtration rate (mGFR) compared with age-matched WT Yucatan pigs (*n* = 4). Unpaired two-tailed Student’s *t*-test; error bars are s.e.m. Points are biological replicates and data are from one experiment. Source Data

**Extended Data Fig. 1:**
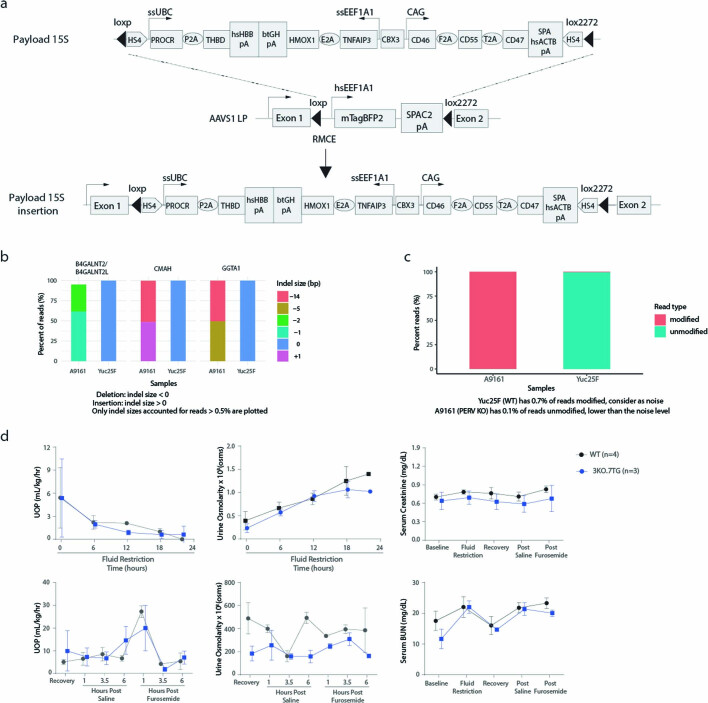
Yucatan porcine donor is engineered to carry 69 genomic edits. **a**, Payload 15 S was inserted into the *AAVS1* genomic safe harbor site by recombinase mediated cassette exchange (RMCE). It carries three transcription units, with the *ssUBC* promoter expressing the *PROCR* and the *THBD* cDNAs, the *ssEEF1A1* promoter expressing the *TNFAIP3* and the *HMOX1* cDNAs, and the *CAG* promoter expressing the *CD46*, *CD55*, and *CD47* cDNAs. The transgenic construct is flanked by the *loxP*/*lox2272* sequences and replaced the landing pad sequence flanked by the same *lox* sites, in an RMCE reaction. **b**, Indel patterns for the *B4GALNT2*/*B4GALNT2L*, *CMAH*, and *GGTA1* genes carried in the porcine donor A9161, analyzed from Amplicon sequencing. **c**, Region encompassing the catalytic core of the reverse transcriptase (RT) was amplified by a PCR reaction and sequenced, from the porcine endogenous retroviral sequences of donor A9161. Compared with the PCR product amplified from the wild type Yuc25F cells, those amplified from A9161 had been modified and predicted to obliterate the RT activity. **d**, Kidneys from 3KO.7TG donors (*n* = 3) responded to fluid challenges in vivo, including fluid restriction, saline bolus challenge, and furosemide (1 mgkg^−1^), similar to WT Yucatan (*n* = 4) kidneys. Data are from one independent experiment. Source Data

The pigs were engineered to carry 69 genomic edits, using the clustered regularly interspaced short palindromic repeats (CRISPR) and CRISPR-associated protein 9 (Cas9)-mediated nonhomologous end joining and homology-directed repair^30,31^, and recombinase-mediated cassette exchange^32^ (Fig. 2a, Extended Data Fig. 1a and Supplementary Table 2). These edits disrupted the three glycan synthesis genes (eight alleles; Extended Data Fig. 1b) (3KO), had a transgenic construct (referred to as Payload 15S (PL15S)) inserted hemizygously into the *AAVS1* site (Extended Data Fig. 1a) (7TG), and inactivated the PERV elements (59 copies) (RI) (Extended Data Fig. 1c) carried in the Yucatan female cells Yuc25F.

PL15S carries seven human genes (Supplementary Table 3), including *CD46* and *CD55* from the complement cascade, *THBD* and *PROCR* from the coagulation pathway, *CD47*, which is involved in innate immunity, and *TNFAIP3* and *HMOX1*, which dampen ischaemia–reperfusion injury, apoptosis and inflammation. The transgenic construct was configured into a polycistronic design, in which two complementary DNA sequences were linked with a viral 2A sequence^33^ and the seven complementary DNAs split among three transcription cassettes (Extended Data Fig. 1a).

Next-generation sequencing was performed on the edited cells and/or the cloned pigs produced after each round of editing and cloning. Here we present data produced from the porcine donor, A9161, carrying the 3KO.7TG.RI genotype, whose kidney was transplanted into NHP recipient M6521 and achieved a graft survival time of 176 days. Long-read whole-genome sequencing showed that one copy of the intact PL15S sequence was inserted into intron 1 of the porcine *PPP1R12C* gene (orthologous to the human *AAVS1* gene) (Fig. 2b, top). Direct RNA sequencing (dRNA-seq) of an A9161 kidney sample indicated that the three transcription units carried in PL15S were transcribed, with the expected transcription initiation, intron excision and mRNA polyadenylation (Fig. 2b, bottom). Among the three units, expression of the *CAG* and *ssUBC* units were higher, whereas expression of the *ssEEF1A1* unit was at a lower level. The 3KO and RI genotypes were verified by sequencing of the PCR products encompassing the CRISPR–Cas9 target sites (Extended Data Fig. 1b,c and Supplementary Table 4).

**Extended Data Fig. 2:**
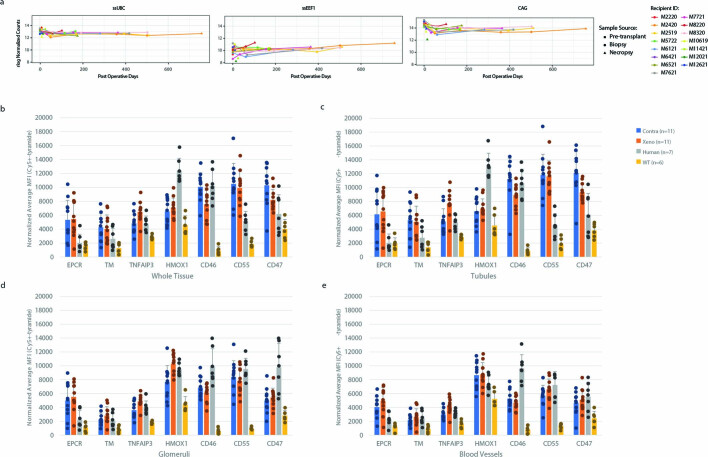
Human transgenes are expressed. **a**, RNA was extracted from contralateral, biopsy, and necropsy kidney samples (3KO.7TG, *n* = 8; 3KO.7TG.RI, *n* = 7) and sequencing performed on the Illumina platform. Transgene expression from each of the three transcription units, *ssUBC*, *ssEEF1A1*, and *CAG*, was analyzed. **b**, Human transgenic protein expression, as detected by IHC, was quantitated from contralateral and necropsy kidney samples for completed NHP transplant studies, except for NHP recipient M7721 (porcine donor 21405), for which a contralateral kidney sample was not available (3KO.7TG, *n* = 6; 3KO.7TG.RI, *n* = 5). **c–e**, Transgenic protein expression was quantitated from tubular cells, glomeruli, and blood vessels, respectively. Each dot represents data from an independent kidney sample. Source Data

RNA-seq and immunohistochemistry (IHC) were performed for all completed 3KO.7TG ± RI renal transplants, except for donor 21405 whose contralateral kidney biopsy sample was not available (*n* = 11). For RNA-seq, contralateral, biopsy and necropsy samples were analysed and showed that all human transgenes were expressed and, again, those under the *CAG* and *ssUBC* promoters were at a higher level, whereas those under the *ssEEF1A1* promoter were at a lower level (Extended Data Fig. 2a). For IHC analysis (Supplementary Table 5), two samples from each renal transplant experiment, the contralateral kidney collected at transplant and the transplanted kidney procured upon necropsy, were analysed. As an example, IHC images from A9161 are shown (Fig. 2c). All seven transgenic proteins were detected in the contralateral kidney, in both the glomeruli and the tubular cells, and expression was maintained in the renal graft at necropsy on post-transplantation day 176. The IHC photomicrographs from all completed NHP studies were scanned and quantified (Extended Data Fig. 2b–e), which showed that all transgenic proteins were detected. Therefore, we conclude that transgene expression was durable.

Contralateral kidney tissues from two porcine donors, A9161 and 21077 (a 3KO.7TG donor), were dissociated into single cells, and single-cell RNA-seq was performed. Three KEC types were identified, including endothelial cells (PECAM1^+^PLVAP^−^EHD3^−^GATA5^−^), fenestrated endothelial cells (PECAM1^+^PLVAP^+^) and glomerular endothelial cells (PECAM1^+^EHD3^+^GATA5^+^) (Fig. 2d). Transcripts from the *CAG* and *ssUBC* cassettes were readily detected in the three endothelial cell types, whereas *ssEEF1A1* cassette transcripts were found in endothelial cells and fenestrated endothelial cells, but not in glomerular endothelial cells. This probably reflects the lower expression of the *ssEEF1A1* cassette shown by direct RNA-seq (Fig. 2b). Nonetheless, both the human TNFAIP3 and HMOX1 proteins were detected in kidneys by IHC (Fig. 2c), and TNFAIP3 was detected by western blot (Extended Data Fig. 5c).

Given the relatively large number of genomic edits carried by the porcine donors, we wanted to see whether kidney function was compromised. The measured glomerular filtration rate of 3KO.7TG donors was not different from age-matched and gender-matched WT Yucatan pigs (Fig. 2e). Furthermore, when subjected to fluid challenge studies, both groups responded to the challenges similarly, providing further evidence that the 3KO.7TG kidneys functioned normally (Extended Data Fig. 1d).

## Genomic edits confer protection

**Extended Data Fig. 3:**
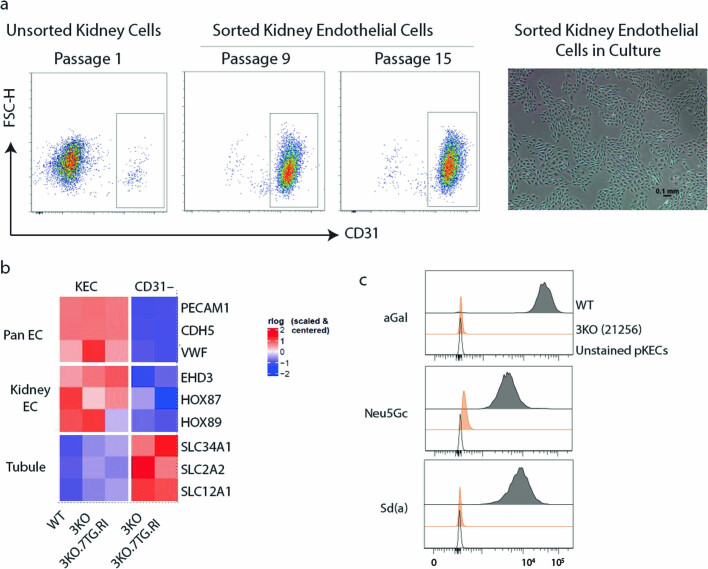
Porcine kidney endothelial cells are derived and characterized. **a**, Kidney endothelial cells (KECs) were enriched by sorting for CD31+ cells twice. Dissociated cells from a kidney preparation contained a low percentage of CD31+ cells (2%). A subsequent second sort substantially enriched the percentage of CD31+ cells. CD31 expression was maintained over passaging as shown with passages 9 and 15 cells. KECs were isolated and enriched CD31 expression was assessed for lines from  41 independent kidneys. (Right Panel) A 40X phase-contrast image of double sorted KECs is provided. **b**, CD31+ KECs expressed endothelial marker genes, analyzed by RNA-seq. **c**, Compared to WT KECs, 3KO KECs lacked the three glycans, analyzed by flow cytometry.

**Extended Data Fig. 4:**
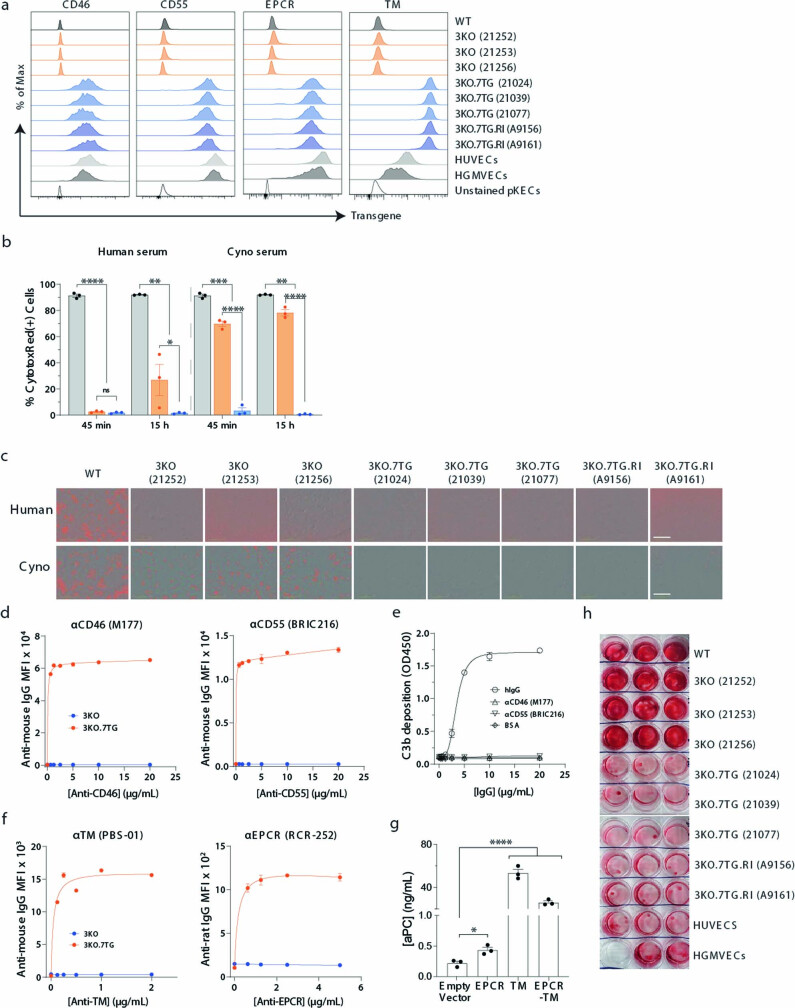
Human CD46, CD55, EPCR, and TM proteins are expressed and functional. **a**, Human CD46, CD55, EPCR, and TM proteins were detected on the surface of 3KO.7TG ± RI KECs. **b**, WT (*n* = 3) and 3KO (*n* = 3) KECs were lysed, whereas the 3KO.7TG KECs (*n* = 3) showed significantly reduced complement dependent cytotoxicity (CDC), when incubated with human or cynomolgus monkey serum over an extended period of time. **c**, Representative images from **3b**, collected from an Incucyte after fluorescent dye staining. Scale bar (in white): 400 μm. **d**, Anti-human CD46 (clone M177, left) and CD55 (clone BRIC216, right) antibodies specifically bound human CD46 or CD55, as demonstrated by the lack of binding to 3KO KECs (*n* = 1, blue line), compared to the 3KO.7TG KECs (n = 1, orange line). **e**, M177 and BRIC216 antibodies did not activate complement fixation compared to human IgG (hIgG). **f**, Similarly, anti-human TM (clone PBS-01) and EPCR (clone RCR-252) antibodies are human specific. (3KO, n = 1; 3KO.7TG, *n* = 1). For **d-f**, average of 2 technical replicates. **g**, EPCR, TM, or both expressed in KECs contributed to aPC production, with TM showing more significant effect. Points are technical replicates. **h**, Extensive clotting was observed when WT (*n* = 1) and 3KO (*n* = 3) KECs were incubated with human whole blood, while 3KO.7TG (*n* = 3) or 3KO.7TG.RI KECs (*n* = 2) showed minimal clotting, similar to human umbilical vein and human glomerular microvascular endothelial cells (HUVECs and HGMVECs). Samples in the same row are technical replicates. Pictures were taken after plasma or nonclotted blood was removed from wells. For **b,g**, ****P < 0.0001, ***P < 0.001, **P < 0.01, * P < 0.05 by unpaired, two-tailed Student’s t-tests. Source Data

**Extended Data Fig. 5:**
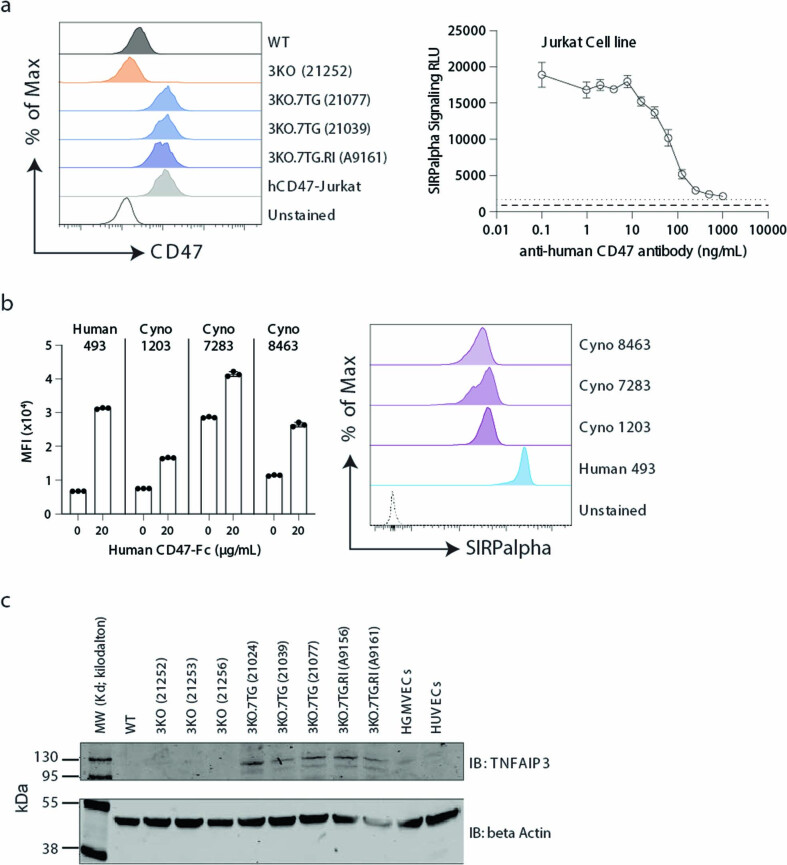
Human CD47 and TNFAIP3 proteins are expressed. **a**, (Left) Human CD47 was detected on 3KO.7TG ± RI KECs and on Jurkat T cells overexpressing human CD47 (hCD47-Jurkat). (Right) In the SIRPα reporter assay, hCD47-Jurkat cells were incubated with SIRPα expressing Jurkat signaling cells, and engagement of hCD47 with human SIRPα produced an activation signal, which was blocked with increasing anti-human CD47 antibodies. Dashed lines: signals of cells alone. Error bars represent standard deviation from mean. **b**, (Left) Recombinant human CD47-Fc fusion protein bound to human (*n* = 1) or cynomolgus monkey (cyno) (*n *= 3) monocytes. Each point represents technical replicates. (Right) SIRPα expression on the monocytes used in the binding assay is shown. Data are representative of two independent experiments. **c**, 3KO.7TG ± RI KECs expressed TNFAIP3 protein as analyzed by Western blot. Uncropped images are provided in Supplementary Fig. 3. Representative data from two independent experiments. Source Data

We isolated primary KECs from pigs (Extended Data Fig. 3a,b) and performed in vitro analysis. As expected, the 3KO KECs lacked α-Gal, Neu5Gc and Sd(a) (Extended Data Fig. 3c) and the 3KO.7TG ± RI KECs expressed human transgenes, as analysed by flow cytometry (Extended Data Fig. 4a, CD46, CD55, EPCR and thrombomodulin; Extended Data Fig. 5a, CD47) and by western blot (Extended Data Fig. 5c, TNFAIP3).

**Fig. 3:**
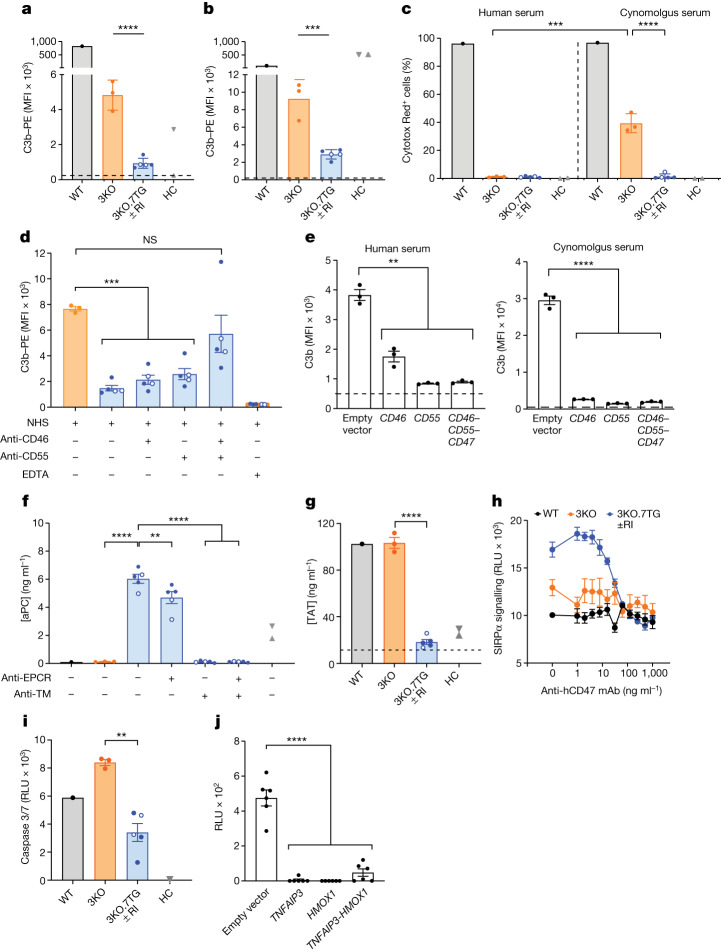
Human transgenes confer protection. **a**,**b**, In human (**a**) or cynomolgus monkey (**b**) serum, deposition of C3b on 3KO.7TG ± RI KECs was further reduced. The dashed lines indicate average C3b deposition (serum + EDTA). **c**, In human or cynomolgus monkey serum, 3KO.7TG ± RI KECs were protected from complement-dependent cytotoxicity. 3KO KECs showed residual complement-dependent cytotoxicity in cynomolgus monkey serum. **d**, The 3KO.7TG ± RI KECs mitigated C3b deposition when human CD46, CD55 or both were not blocked by antibodies. NHS, normal human serum. **e**, 3KO KECs carrying *CD46*, *CD55* or *CD46*–*CD55*–*CD47* polycistronic genes regulated C3b deposition in human or cynomolgus monkey serum. The dashed lines indicate average C3b deposition from EDTA-treated serum. **f**, The 3KO.7TG ± RI KECs readily generated aPC. aPC production was reduced when EPCR or thrombomodulin (TM) was blocked with antibodies, with blocking of thrombomodulin being more effective. The dashed line indicates average aPC production without cells. **g**, 3KO.7TG ± RI KECs regulated TAT formation in whole blood. The dashed line indicates baseline TAT in donor blood. **h**, Human CD47 (hCD47) on 3KO.7TG ± RI KECs signalled through SIRPα on human Jurkat cells. WT KECs (*n* = 1), 3KO KECs (*n* = 1), 3KO.7TG ± RI (*n* = 2 for 3KO.7TG and *n* = 1 for 3KO.7TG.RI). mAb, monoclonal antibody; RLU, relative light unit. **i**, 3KO.7TG ± RI KECs were protected from human TNF-induced caspase 3/7 activation. **j**, 3KO kidney cortex-derived cells expressing *TNFAIP3*, *HMOX1* or *TNFAIP3*–*HMOX1* polycistronic genes were protected from human TNF-induced caspase 3/7 activation. In **a**–**d**,**f**–**i**, WT (*n* = 1), 3KO (*n* = 3), 3KO.7TG (*n* = 3, solid blue circles), 3KO.7TG.RI (*n* = 2, open circles), human controls (HC) and human umbilical vein endothelial cells (*n* = 1, grey triangle). In **a**–**d**,**f**,**g**, human glomerular microvascular endothelial cells (*n* = 1, grey inverted triangle). For **a**–**d**,**f**–**i**, data are from biological replicate, repeated at least twice. For **e**,**j**, data are from a technical replicate. Error bars indicate s.e.m. Statistical analyses were two-tailed, paired (3KO.7TG ± RI with versus without blocking antibodies (**f**)) and unpaired (all other comparisons) Student’s *t*-tests. ****P < 0.0001, ***P < 0.001, **P < 0.01; NS, not significant. Source Data

When incubated in human or cynomolgus monkey serum, the 3KO.7TG ± RI KECs exhibited significantly less C3b deposition than 3KO cells, suggesting that the transgene (or transgenes) impeded complement activation (Fig. 3a,b). When incubated with human or cynomolgus monkey serum for 45 min, WT cells were lysed, whereas the 3KO modifications alone almost completely abolished complement-dependent cytotoxicity of human serum, but not cynomolgus monkey serum (Fig. 3c and Extended Data Fig. 4b,c). This suggests that cynomolgus monkey serum has a stronger anti-porcine cytotoxic activity than human serum. When the incubation time was extended to 15 h, the 3KO modification was insufficient to fully protect the cells even from cell death by human serum, whereas 3KO.7TG ± RI KECs were protected against both human and cynomolgus monkey serum cytotoxicity, beyond the protection afforded by 3KO (Extended Data Fig. 4b). The contribution of the *CD46* and *CD55* transgenes was verified by blocking antibodies (Fig. 3d and Extended Data Fig. 4d,e). Furthermore, when CD46, CD55 or both were expressed in 3KO KECs, each was functionally competent to mitigate complement activation (Fig. 3e). Collectively, these data demonstrate that transgenic human CD46 and CD55 proteins regulate complement activity when expressed on porcine KECs. By including both transgenes, we mimic the naturally built-in redundancy, which may render the system more resilient in the case that one of the regulators is lost by shedding (CD46)^34^ or by enzymatic cleavage (CD55)^35^ during inflammation or tissue injury.

Unlike WT or 3KO porcine cells, 3KO.7TG ± RI KECs readily produced aPC (Fig. 3f), reduced TAT formation (Fig. 3g) and regulated coagulation of human whole blood as efficiently as human control cells (Extended Data Fig. 4h). Using blocking antibodies (Extended Data Fig. 4f), we showed that both EPCR and thrombomodulin contributed to aPC production (Fig. 3f), with thrombomodulin having a more prominent effect. In addition, 3KO KECs overexpressing EPCR, thrombomodulin or both were engineered and showed that both EPCR and thrombomodulin contributed to aPC production, with thrombomodulin having a more significant effect in this experimental system (Extended Data Fig. 4g). Although both thrombomodulin and EPCR contribute to aPC production, their mechanisms of action differ^36^. Thrombomodulin binds to thrombin and functions as a cofactor in the thrombin-induced activation of protein C, whereas EPCR binds to protein C and presents it to the thrombomodulin–thrombin activation complex. Given the synergistic effect of the two proteins, we reasoned that coagulation may be better regulated when both are provided.

Porcine CD47 does not engage the human SIRPα receptor effectively^37^. Using a SIRPα reporter cell line, we found that 3KO.7TG ± RI KECs expressing transgenic human CD47 (Extended Data Fig. 5a) activated the SIRPα signalling pathway, and pre-incubation with the anti-human CD47 antibody blocked SIRPα signalling in a dose-dependent manner (Fig. 3h). In addition, the capacity of human CD47 to engage NHP SIRPα receptors was demonstrated in a binding assay using a human CD47 fusion protein to stain monocytes from cynomolgus monkey expressing endogenous SIRPα (Extended Data Fig. 5b).

Finally, activation of innate immune cells in the xenograft induces inflammation and apoptosis of endothelial cells^13^. To improve tissue resilience, human TNFAIP3 and HMOX1 were expressed in 3KO.7TG ± RI kidneys (Fig. 2c). Western blot analysis confirmed TNFAIP3 expression in 3KO.7TG ± RI KECs (Extended Data Fig. 5c). The effect of transgenic TNFAIP3 and HMOX1 expression on apoptosis was assessed in a caspase 3/7 assay, which showed that transgenic protein levels in 3KO.7TG cells were sufficient to reduce caspase 3/7 activation following human TNF treatment, compared with 3KO KECs (Fig. 3i). Furthermore, kidney cortex-derived cells overexpressing TNFAIP3, HMOX1 or both effectively blocked TNF-induced caspase 3/7 activation in vitro (Fig. 3j). Although overlapping in their anti-apoptotic activities, TNFAIP3 and HMOX1 achieve this outcome by their unique biological functions and dampen inflammation under distinct circumstances. TNFAIP3 is a deubiquitinating enzyme and inhibits TNF-mediated apoptosis^38^, whereas HMOX1 is involved in the initial step of haem degradation and has been shown to have antioxidant and anti-inflammatory effects^38,39^. We included both TNFAIP3 and HMOX1 in our donors, as both transgenes have been shown to provide benefit in vitro and in vivo^40,41^.

## Engineered porcine kidney supports life

**Extended Data Fig. 6:**
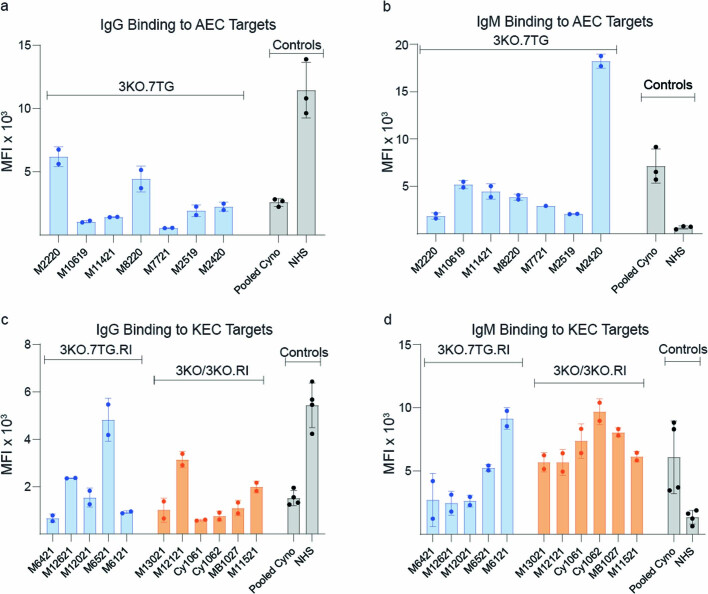
Cynomolgus monkeys were examined for preformed IgG and IgM antibodies. **a**, Pre-transplant serum samples from NHP recipients that received 3KO.7TG renal grafts were incubated with aortic endothelial cells (AECs) derived from a 3KO porcine donor and bound IgG measured. Displayed data were compiled from *n* = 3 independent experiments, with reference controls run in each experiment. For a given sample, each point represents a technical replicate, while points for the two reference controls represent experimental replicates. **b**, Same as in **a** but anti-porcine IgM binding was measured. **c**, Pre-transplant serum samples from NHP recipients that received 3KO.7TG.RI or 3KO ± RI renal grafts were incubated with KECs derived from a 3KO porcine donor and bound IgG measured. Displayed data were compiled from* n* = 4 independent experiments, with reference controls run in each experiment. For a given sample, each point represents a technical replicate, while points for the two reference controls represent experimental replicates. **d**, Same as in **c** but anti-porcine IgM antibody binding was measured. NHS, normal human serum; Pooled Cyno, serum pooled from 96 cynomolgus monkeys. Source Data

A cohort of cynomolgus monkeys was screened for porcine-reactive preformed antibody binding. In general, monkeys with a lower antibody binding to 3KO AECs or KECs were selected (Extended Data Fig. 6a–d).

Given that NHPs are expensive, highly regulated and limited in availability, it was not possible to conduct a statistically powered experiment. Therefore, the number of NHP transplants per porcine donor genotype group was empirically determined, based on historical data reported in literature and what could be reasonably achieved. The pairing of a porcine donor with an NHP recipient was dictated by availability. In addition, the study was not blind for all involved. The recipients were administered an immunosuppression regimen of induction therapy with B and T lymphocyte depletion, maintenance therapy with anti-CD154 antibody and mycophenolate mofetil, and a brief post-transplant course of tacrolimus and steroids (Supplementary Fig. 1). A genetically engineered porcine kidney was transplanted, concurrently with nephrectomy of the two native kidneys of the cynomolgus monkey.

**Fig. 4:**
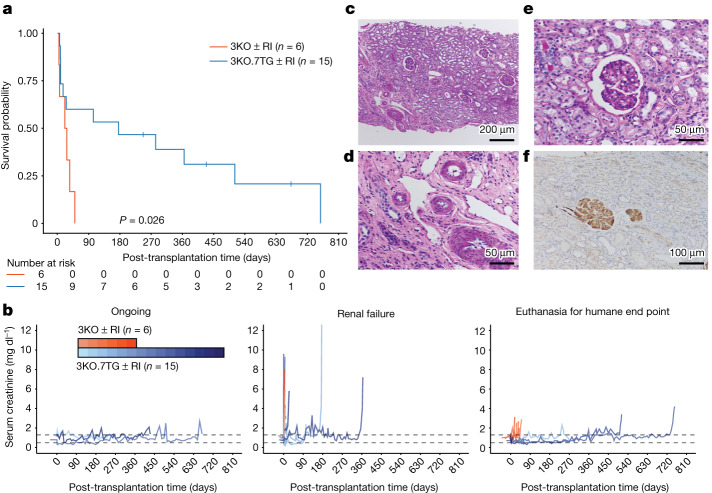
Engineered porcine kidneys support life in cynomolgus monkeys. **a**, Significantly improved survival probability (*P* = 0.026) for recipients transplanted with 3KO.7TG ± RI renal grafts (*n* = 15) compared with 3KO ± RI (*n* = 6). Statistical analysis was performed using a log-rank test. **b**, Serum creatinine levels generally remained within normal range, except when associated with graft failure. Creatinine levels above 6 mg dl^−1^ meet criteria for euthanasia. Dashed lines indicate normal range of serum creatinine levels. **c**, Periodic acid–Schiff staining of a kidney biopsy sample derived from recipient M2420, carrying a 3KO.7TG renal graft, at POD 502, appears normal. **d**,**e**, Blood vessels (**d**) and glomerulus (**e**) of M2420 renal graft biopsy at POD 502 appear normal. **f**, C4d-positive staining in glomeruli, but not in peritubular capillaries, was observed in M2420 renal graft biopsy at POD 502. Source Data

Given the similar performance of KECs isolated from 3KO.7TG or 3KO.7TG.RI donors in functional assays (Fig. 3 and Extended Data Figs. 3–5), we analysed transplants performed with 3KO kidneys with and without RI as one group and 3KO.7TG and 3KO.7TG.RI kidneys as a second group. Survival of the six 3KO ± RI kidney transplant recipients was short, with end of study at days 4 and 6 (renal failure), 21 (disseminated intravascular coagulation), and 26, 35 and 50 (severe oedema and proteinuria) (Table 1). By contrast, the 3KO.7TG (*n* = 8) and 3KO.7TG.RI (*n* = 7) transplants achieved significantly longer graft survival than the 3KO ± RI xenografts (*n* = 6) (median survival time of 176 days versus 24 days; *P* = 0.026, log-rank test) (Table 1 and Fig. 4a). The filtration of metabolites, such as creatinine, by the single transplanted porcine kidney was sufficient to compensate for the lack of two native kidneys (Fig. 4b), as observed routinely in human renal allotransplantation. Other parameters, including serum albumin, serum potassium and blood platelet counts, generally remained within normal range, except when associated with renal failure (serum albumin and potassium) (Supplementary Fig. 5).

**Table 1:** Renal grafts support life in pig-to-NHP xenotransplantation

Genotype	Animal ID	Survival (days)^a^	Reason for euthanasia^b^	EOS	Xenograft injury patterns	De novo DSA^c^
3KO.RI	M13021	4	Renal failure	–	ATI	No
	M12121	6	Renal failure	T	AMR, TMA	Yes
	CY1061^d^	21	HE — intra-abdominal haemorrhage, DIC with refractory anaemia and renal insufficiency	I, C/A, T, U	ATI, TMA	No
	CY1062^d^	26	HE — respiratory insufficiency with haemoptysis, peripheral oedema and renal insufficiency	I, C/A	ATI, TMA	No
	MB1027^d^	35	HE — peripheral oedema and renal insufficiency	–	ATI, TMA	Yes
3KO	M11521^e^	50	HE — peripheral oedema	C/A, T, BH	ATI, TCMR-IA	Yes
3KO.7TG^f^	M2220	8	HE — respiratory insufficiency with pleural effusions and renal insufficiency	–	AMR, TMA	No
	M10619	9	Renal failure	C/A, T	AMR, TMA	No
	M11421	25	Renal failure	BH	AMR, ATI, TMA	No
	M8220^g,h^	103	Renal failure	I, BH	CAMR, TCMR-III, TMA	Yes
	M7721	365	Renal failure	C/A	CAMR, TMA	Yes
	M2519	511	HE — epistaxis with refractory anaemia and renal insufficiency	I, C/A, T	TMA	Yes
	M2420	758	HE — peripheral oedema and renal insufficiency	I, C/A	CAMR, TMA	Yes
	M8320	>673, ongoing	–	–	–	NC
3KO.7TG.RI^f^	M6421^g,i^	6	Renal failure	T	ATI, TMA	No
	M12621^g,i^	9	Renal failure	–	AMR, ATI, TMA	No
	M12021	16	Renal failure	I	TMA	No
	M6521^h^	176	Renal failure	C/A	CAMR, TMA	Yes
	M6121	283	HE — acute paraplegia	–	No evidence of rejection	No
	M5722^g,i^	>247, ongoing	–	–	–	NC
	M7621	>429, ongoing	–	–	–	NC

3KO, three knockout (*GGTA1*, *CMAH* and *B4GALNT2/B4GALNT2L*); 7TG, seven human transgenes (*CD46*, *CD55*, *THBD*, *PROCR*, *CD47*, *TNFAIP3* and *HMOX1*); >, beyond; –, not present; AMR, antibody-mediated rejection; ATI, acute tubular injury; BH, body habitus including weight and skin condition; C/A, coagulopathy or refractory anaemia; CAMR, chronic active AMR; DIC, disseminated intravascular coagulation; DSA, donor-specific antibody; EOS, end of study; HE, humane end point; I, suspected or confirmed infection; NC, not completed; RI, retroviral inactivation (PERV knockout); T, thrombocytopenia (<100 × 10^6^ platelets per millilitre); TCMR-IA, T cell-mediated rejection grade IA; TMA, thrombotic microangiopathy; U, ureteral stricture managed by redo ureterovesical anastomosis.

^a^Survival days were based on data cut-off on 31 March 2023. ^b^EOS based on renal failure alone defined by multiple creatinine values of more than 6–8 mg dl^−1^ and/or BUN (blood urea nitrogen) of more than 100 mg dl^−1^ unless noted as HE. Renal insufficiency denotes creatinine values of 3–6 mg dl^−1^ and/or BUN 60–100 mg dl^−1^, which alone did not meet criteria for euthanasia, but in the context of severe symptomatology, met criteria for HE. ^c^Denotes DSA (IgM or IgG) in peripheral blood (Extended Data Fig. 8). ^d^Denotes transplants carried out at additional sites (Duke University for MB1027 and University of Wisconsin for CY1061 and CY1062). ^e^In addition to 3KO, this xenograft also carries a ‘landing pad’ sequence inserted at the *AAVS1* safe harbour site. ^f^3KO.7TG and 3KO.7TG.RI are also referred to as EGEN-2734 and EGEN-2784, respectively, in other publications. ^g^Denotes animals receiving co-stimulation blockade with TNX-1500. ^h^Denotes recipients who had only unilateral native nephrectomy at time of transplantation, with the remaining native nephrectomy performed around post-operative day 20. ^i^Denotes animals that did not receive MMF (mycophenylate mofetil) as part of their maintenance immunosuppression.

Rejection was assessed by histopathological examination, based on the Banff classification of renal allograft pathology^42^, with modifications for xenotransplantation (Table 1 and Extended Data Table 1). Five out of the six 3KO ± RI kidneys showed evidence of acute tubular injury (ATI), a feature not included in the Banff criteria but representing general kidney injury. In addition, antibody-mediated rejection (AMR)/thrombotic microangiopathy (TMA) (M12121), TMA (CY1061, CY1062 and MB1027) and T cell-mediated rejection (TCMR) (M11521) were also observed. In 3KO.7TG ± RI kidney recipients, those who developed renal failure exhibited TMA with or without AMR at time of euthanasia and only two showed evidence of ATI. Unlike allotransplantation, T cell-mediated rejection was not a major pathology in xenotransplantation, with only one graft loss meeting Banff criteria for TCMR. It remains to be determined whether the current Banff scoring system developed for allotransplantation in humans can be satisfactorily applied to xenotransplantation, as rejection mechanisms may differ^43^. In allotransplantation, TMA usually reflects an antibody-mediated or drug-related process, whereas in xenotransplantation, substantial species coagulation or complement regulatory protein incompatibility may contribute to TMA, even in the absence of de novo donor-specific antibody (dnDSA). With these considerations, pathology samples were assessed with a ‘xenotransplantation-adapted’ criteria (Extended Data Table 1 and Supplementary Table 6). These xenotransplantation-adapted criteria represent our effort at informative and useful scoring and diagnosis in renal xenotransplantation.

**Extended Data Fig. 7:**
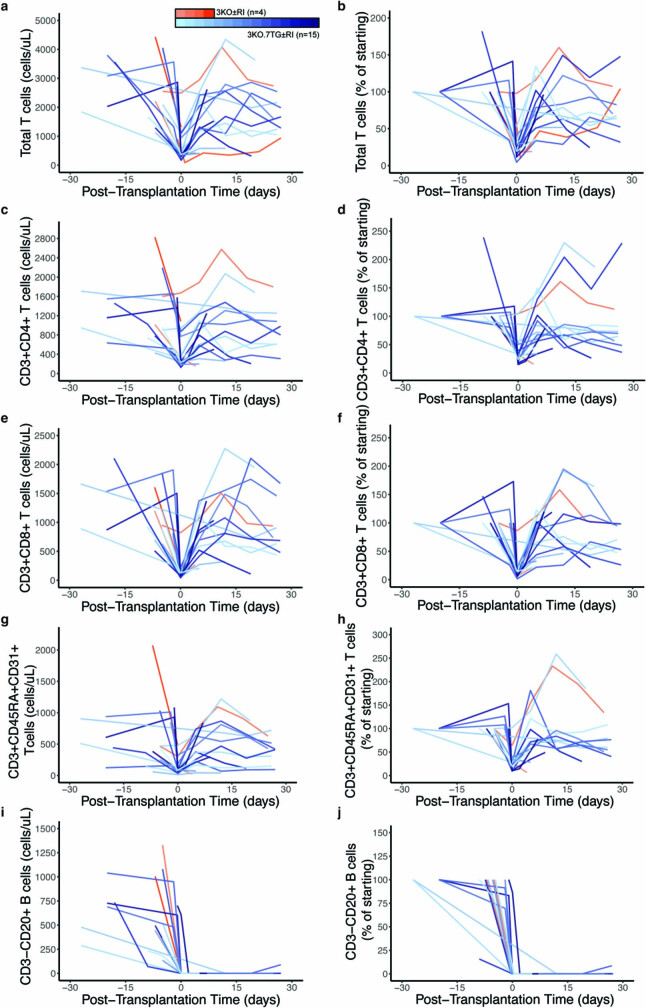
Lymphocytes were depleted following induction immunosuppression. Flow cytometry demonstrated depletion of circulating immune cells with rhATG and anti-CD20 mAb. Absolute counts of immune cells pre-transplant (left panels) and relative changes as compared to the first pre-transplant measurement (right panels) for total T cells (**a**,**b**), CD3 + CD4 + T cells (**c,d**), CD3 + CD8 + T cells (**e**,**f**), CD3 + CD45RA + CD31+ recent thymic emigrant T cells (**g**,**h**), and CD3-CD20 + B cells (**i**,**j**). Source Data

**Extended Data Fig. 8:**
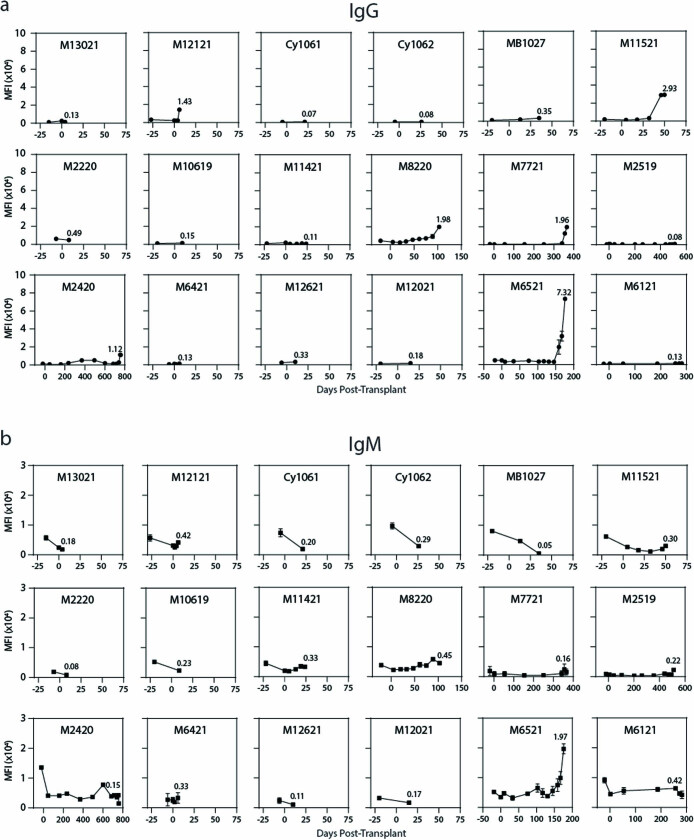
Development of de novo anti-porcine IgG and IgM antibodies. **a**, Pre- and post-transplant serum samples from NHP recipients that received 3KO ± RI or 3KO.7TG ± RI renal grafts were incubated with aortic or kidney derived endothelial cells isolated from a 3KO porcine donor and bound IgG measured across 2 technical replicates for each sample. **b**, Same as in **a** but anti-porcine IgM binding was measured. For **a** and **b**, data are compiled from seven independent experiments, where all samples from a given recipient were screened in the same experiment. Source Data

**Extended Data Fig. 9:**
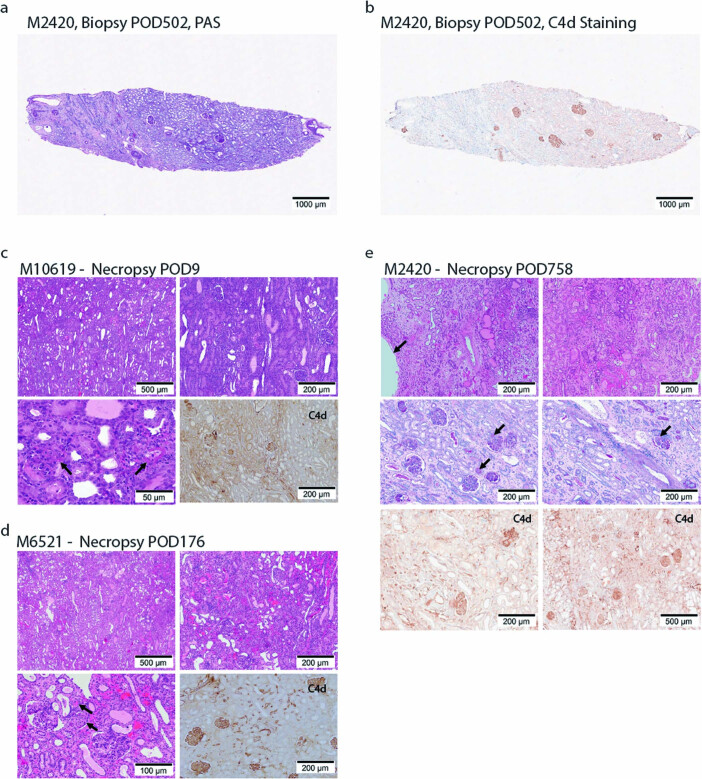
Histological evaluation of transplanted kidney samples. **a** & **b**, whole kidney biospy section for recipient M2420 at post-operative day (POD) 502 (see related high power images in Fig. 4f–i). Whole slide scan of a PAS (periodic acid-Schiff) stained section demonstrates a focal scar at left with preserved parenchyma at middle and right (a) and stained with anti-C4d antibody and visualized with horse-radish peroxidase shows predominantly glomerular C4d and peritubular capillary C4d score of 0 by Banff (b). **c**, **d**, & **e**: Histopathologic photomicrographs from necropsy samples of xenografts isolated from M10619 (POD9), M6521 (POD176), and M2420 (POD758) respectively. Kidney sections were stained with H&E (haematoxylin and eosin) and C4d (**c**, **d**) or H&E, PAS, and C4d (**e**) and photomicrographs of various magnifications taken. The C4d Banff scores for the 3 xenokidneys are 2, 3, and 3 respectively.

Lymphocyte depletion in blood was demonstrated by measuring circulating B cell and T cell counts (Extended Data Fig. 7). Although B cell depletion was robust, dnDSA reactive to 3KO endothelial cells was detected in some animals over time (8 of 18 transplants; Table 1 and Extended Data Fig. 8) and pathological features of antibody-mediated graft injury were observed. For recipient M2420 with the longest xenograft survival time (758 days), a protocol biopsy showed mostly normal kidney histology at day 502 after transplant, with patchy fibrosis (Fig. 4c–e and Extended Data Fig. 9a). However, C4d staining was observed in the glomeruli, suggesting C4 activation (Fig. 4f and Extended Data Fig. 9b). C4 activation is upstream of CD46 and CD55 intervention and positive C4d staining may be expected in the 3KO.7TG ± RI kidney samples in the presence of dnDSA. Representative histopathological photomicrographs from necropsy samples with survival times of 9 (M10619), 176 (M6521) and 758 (M2420) days are provided in Extended Data Fig. 9c–e. Among the long-term survivors, simple appearing, benign cysts in the kidney were observed. Their aetiology is unknown and currently under investigation.

In NHPs with dnDSA, it is possible that the current immunosuppressive regimen was initially efficacious but eventually failed to prevent the development of porcine-specific humoral responses. TMA and AMR in the long-term survivors were often associated with infections or biopsy procedures, which might have triggered an immune response. Although these observations can inform the design of immunosuppressive regimens for clinical studies (for example, consideration of depletion with an anti-CD19 antibody and desensitization or salvage therapy with plasmapheresis or targeted plasma cell therapies), approaches that can be applied in the NHP model are limited. Of note, agents targeting the CD40–CD40L pathway have become standard of care in NHP xenotransplantation studies. Although these agents have not received FDA approval, several are in clinical development and will serve as a cornerstone in the immunosuppressive regimen, along with FDA-approved drugs currently used in clinical kidney transplantation.

## Discussion

In this study, we describe a porcine donor carrying 69 genomic edits, with expression and function of all 7 human transgenes. Although a porcine donor carrying a *CMAH* knockout is thought not to survive in OWMs considering complication from a *CMAH* knockout^8,9^, renal grafts derived from the 3KO.7TG ± RI porcine donor supported life long-term in cynomolgus monkeys, up to 758 days.

The variable graft survival time may be inherent to kidney xenotransplantation and/or unique to the OWM model. Although 3KO substantially reduced the level of pre-formed anti-porcine antibody binding, minor xenoantigens remain and contributed to residual antibody binding (Fig. 1b). Furthermore, complement-dependent cytotoxicity activity was higher in cynomolgus monkey serum than in human serum (Fig. 3c and Extended Data Fig. 4b,c). In addition, data suggest that *CMAH* inactivation may produce a novel antigen xenogenic to the OWMs, which may be a target of preformed antibody binding, leading to complement activation in OWM serum^8,9^. It is worth noting that in a clinical setting, the complication around *CMAH* knockout unique to the OWMs will not be relevant, as it will be a match with the *CMAH* pseudogene genotype of the human recipient.

One advantage of xenotransplantation, as compared with allotransplantation, is the opportunity to genetically engineer a donor organ. Therefore, to promote graft survival, the burden may be shifted from a heavy immunosuppression regimen on the recipient to a more optimal graft donated from a genetically engineered porcine donor. Given the substantial molecular incompatibilities between the two species, the inclusion of additional human transgenes may be considered, such as *TFPI*, *CD39* (also known as *ENTPD1*), *HLA-E* and *PDL1* (also known as *CD274*)^14,44^. To recapitulate the pattern and level of expression of the porcine endogenous genes, in situ knock-in, in which the human coding sequence replaces the porcine orthologous gene, could be considered. This is particularly pertinent for *THBD* and *PROCR*, which are normally expressed in endothelial cells. We recognize that in addition to the glycan antigens, a vast array of porcine proteins may also be xenogenic^17^ and it may not be possible to identify and eliminate them all. Ultimately, a genetically engineered porcine model, with an immune tolerance feature^45^, may be the goal.

The successful proof-of-principle study achieved in this study brings us closer to clinical testing of porcine renal grafts for human transplantation.

## Methods

### Assembly of transgenic constructs

The landing pad construct carries the human *EEF1A1* promoter driving expression of the *mTagBFP2* marker gene, flanked by a pair of *loxP*/*lox2272* sites. The left homology arm of 1,469 bp (chromosome 6 coordinates, from 59,347,343–59,345,875, susScr11) and the right homology arm of 1,260 bp (chromosome 6 coordinates, from 59,345,874 to 59,344,615, susScr11) were amplified from genomic DNA isolated from the Yucatan breed and placed 5′ or 3′ to the *loxP*/*lox2272* sites. The PL15S transgenic construct was assembled by yeast homologous recombination^46^. In brief, the human coding DNA sequences (Supplementary Table 3), promoter, terminator and polyadenylation sequences were arranged into one of the three polycistronic transcription units, which were further arranged into a linear DNA molecule in a convergent or divergent configuration (Extended Data Fig. 1).

### CRISPR–Cas9 guide RNA design

The R library DECIPHER^47^ was used to design guide RNAs targeting the sequence encoding the catalytic core of the *pol* enzyme from the PERV element (Supplementary Table 2). To inactivate the four genes involved in synthesizing the three major glycan antigens, α-Gal, Neu5Gc and Sd(a), we used one single guide RNA (sgRNA) per gene, targeting the *GGTA1*, *CMAH* or *B4GALNT2/B4GALNT2L* gene. To insert the landing pad DNA into the *AAVS1* genomic locus, we used one guide RNA targeting the *AAVS1* locus. Guide sequences are provided in Supplementary Table 2. All sgRNAs were synthesized and provided by Synthego.

### CRISPR–Cas9-mediated nonhomologous end joining and homology-directed repair mutations

An sgRNA was incubated with the Cas9 enzyme (A36496, Thermo Fisher) to form the ribonucleoprotein (RNP) complex immediately before use, according to the manufacturer’s instructions. To elicit knockouts of the three xenoantigen genes and insertion of the landing pad in a multiplexed reaction, the *AAVS1* landing pad donor plasmid was added to the RNP complexes before electroporation. To inactivate the PERV elements, the three sgRNAs were complexed with the Cas9 protein to form RNPs. Electroporation was performed with the Neon Transfection System 100 µl Kit (MPK10096, Thermo Fisher).

### Generation of the porcine donors carrying 3KO, PL15S insertion into the *AAVS1* site (7TG) and RI

A total of 1 × 10^6^ ear-punch-derived cells (EPDCs) were electroporated (MPK10096, Thermo Fisher) with the four RNPs targeting the *GGTA1*, *CMAH* and *B4GALNT2/B4GALNT2L* genes, and the *AAVS1* locus, together with the *AAVS1* landing pad donor plasmid, and five days later, cells were sorted into single cells gated on the absence of the α-Gal glycan (isolectin B4, FITC conjugate, ALX-650-001F-MC05, Enzo Life Sciences) and the presence of the *mTagBFP2* marker gene and placed into individual wells of a 96-well plate. Clonal populations of the cells were subsequently genotyped to identify those that carried the correct edits of 3KO and successful landing pad insertion into the *AAVS1* site (Supplementary Table 4). Edited cells were then used as nuclear donors in a somatic cell nuclear transfer (SCNT) experiment to produce pigs carrying these edits.

EPDCs carrying 3KO and the landing pad inserted at the *AAVS1* site were isolated and electroporated with the PL15S transgenic construct, along with the *Cre* recombinase mRNA (130-101-113, Miltenyi Biotec), to enable recombinase-mediated cassette exchange. Subsequently, cells were sorted into single cells gated on cell-surface expression of the genes carried on the payload (*CD46*, *CD55*, *PROCR*, *THBD* or *CD47*), and placed into single wells of a 96-well plate. Clonal populations of cells were genotyped to identify those that carry successful replacement of the landing pad with the PL15S sequence (Supplementary Table 4). These edited cells were used in an SCNT experiment and cloned into pigs.

To determine the number of copies of the PERV elements carried in the Yucatan genome, droplet digital PCR was performed as previously described^48^. Analysed with an assay designed against the *pol* gene, the Yucatan 25 female line (Yuc25F) was found to carry 59 copies of the sequence. To inactivate the PERV elements, EPDCs carrying 3KO and PL15S inserted at the *AAVS1* site were derived and electroporated with the three RNPs targeting the catalytic core of the reverse transcriptase activity of the *pol* gene (Supplementary Table 2), and single cells were sorted into single wells of a 96-well plate. The clonal populations of cells were genotyped by amplicon sequencing (Supplementary Table 4) and those carrying indel mutations on all copies of the PERV elements were identified. The edited cells were used in an SCNT experiment and cloned into pigs.

### Porcine donor production by somatic cell nuclear transfer

Gene-edited cells were cloned into pigs by SCNT^49^ and cloning was performed by eGenesis Wisconsin and Precigen Exemplar. Animal cloning was performed under Institutional Animal Care and Use Committee-approved protocols (eGenesis Wisconsin protocol HF2020-01, approved 24 November 2020; Precigen Exemplar protocol MRP2018-003, approved 21 June 2018). All resulting porcine Yucatan donors (*Sus scrofa domesticus*) were female Yucatans. All donor production strictly followed the Guide for the Care and Use of Laboratory Animals (National Research Council of National Academies), particularly the 3R principles.

### IHC staining and analysis of transgene expression

Expression of the human proteins (EPCR, thrombomodulin, TNFAIP3, HMOX1, CD46, CD55 and CD47) was assessed from formalin-fixed, paraffin-embedded tissue sections of 8-week-old WT and transgenic Yucatan porcine kidney samples based on standard (tyramide signal amplification) protocols using Cy5^+^-tyramide as detection reagent. After all targets were detected, tissue sections were counterstained with Hoechst 33258 and mounted with ProLong Glass antifade mountant. Stained tissue sections were imaged using a Zeiss Axio Scan.Z1 automated whole-slide fluorescence scanner using the same scanning parameters for each tissue. Images were generated using the Zeiss Zen Blue 3.4 image analysis software. A list of reagents is provided in Supplementary Table 5.

Mean fluorescent intensities (MFIs) of the transgene proteins in the whole kidney tissues, glomeruli, tubules and blood vessels of positive and negative controls were measured using the Zeiss ZEN Blue 3.4 image analysis software. Positive controls consisted of samples stained with primary antibodies specific to the human transgene proteins, goat anti-mouse–horseradish peroxidase (HRP) or goat anti-rabbit–HRP conjugate and Cy5^+^-tyramide. Negative controls consisted of samples incubated with the mouse–HRP or rabbit–HRP conjugate and Cy5^+^-tyramide only.

Whole-tissue and tissue biopsy measurements were done by drawing around the contour of the entire tissue. The MFI values correspond to the pixel intensities inside the contour as calculated by the software. For the glomerular and blood vessel MFI measurements in whole tissue, 20 glomeruli and 8 blood vessels were selected randomly with equal distribution within the tissue, and contours were drawn to define the structures. For small tissue biopsies, 5–10 glomeruli and 3–5 blood vessels were selected. Average MFI values for the glomeruli and blood vessels (per whole tissue or tissue biopsy) were calculated. Tubular MFI measurement was done using 20 rectangles (measured by the software as 500 × 500 pixels) and 5–10 rectangles (measured by the software as 300 × 300 pixels) in whole tissue and tissue biopsy, respectively. The average MFI values for the 20 rectangles (per tissue) or 5–10 rectangles (per tissue biopsy) were calculated. The reported MFI values in the bar graphs correspond to the normalized average MFI (that is, specific signal) of the 11 samples. Normalized average MFI is defined as the average MFI values of the positive controls minus the average MFI values of the negative controls (that is, nonspecific signal).

### IHC staining for 3KO in kidneys

Formalin-fixed, paraffin-embedded tissue sections of kidney samples from Yucatan porcine, human, cynomolgus, rhesus and baboon were processed as described above using the 1X Thermo Fisher citrate buffer for heat-induced epitope retrieval in a pretreatment (PT) module. After heat-induced epitope retrieval, tissue sections were blocked in TBS plus 5% goat serum for 30 min followed by a 2-h incubation with a mixture of binding reagents in TBS with 5% goat serum. The binding reagent mix consisted of 1:100 dilution of isolectin B4-FITC (detecting the α-Gal antigen), 1:100 dilution of chicken anti-Neu5GC antibody (detecting the Neu5GC antigen) and 1:250 dilution of DBA-biotin (detecting the Sd(a) antigen). Tissue sections were washed with TBS-T three times for 3 min each time and incubated with a mixture of a 1:1,000 dilution of goat anti-chicken Alexa Fluor 647 and a 1:1,000 dilution of streptavidin-Alexa Fluor 568 in TBS supplemented with 5% goat serum. Nuclear staining, tissue mounting with ProLong Glass antifade mountant, imaging and image analysis were performed as described above.

### Measured glomerular filtration rate analysis

Four-month-old WT and the 3KO.7TG Yucatan swine received a single intravenous dose of Omnipaque 300 Iohexol at 64.7 mg kg^−1^ (00407141359, GE Healthcare). Blood samples were collected at 5, 15 and 30 min and 1, 2, 3, 6, 8 10 and 24 h post-dosing, and plasma was separated using K_2_EDTA. Iohexol concentrations were measured using high-performance liquid chromatography with ultraviolet spectroscopy (HPLC-UV). Various pharmacokinetic parameters including clearance (ml min kg^−1^) were calculated using a two-compartmental model (Phoenix WinNonlin, version 8.1 software). Body surface area (BSA) was calculated as BSA (m^2^) = 9 × BW^2/3^/100, and measured glomerular filtration rate (ml min m^−^^2^) was calculated as Iohexol clearance normalized to BSA^50^. Data were plotted and statistics were calculated using GraphPad Prism v8.2.0.

### Primate anti-porcine IgG and IgM analysis

Endothelial cells from 3KO porcine animals without human transgenes were used to measure anti-porcine IgG and IgM antibodies in heat-inactivated serum obtained from cynomolgus recipients before and after transplantation, along with pools of cynomolgus serum from 96 animals, and human serum from at least 100 healthy donors (SeraCare Life Sciences). Each endothelial cell sample (1 × 10^5^ cells per test) was incubated with serum diluted 1:4 in 1× PBS with 1% BSA at 4 °C for 30 min. Cells were washed and incubated at 4 °C for 30 min with Alexa Fluor 488 conjugated F(ab′)2 anti-human IgG (109-546-098, Jackson ImmunoResearch) or Alexa Fluor 647 conjugated F(ab′)2 anti-human IgM (109-606-129, Jackson ImmunoResearch) secondary antibody diluted 1:100. The samples were fixed in 4.2% paraformaldehyde, acquired within 3 days on a FACSymphony A3 (BD Bioscience) and analysed using Flow Jo software v10.6.1 (Flow Jo LLC). MFI levels of IgG and IgM were evaluated in duplicate. MFI data were plotted and statistics were calculated using GraphPad Prism v8.2.0.

### C3b deposition assay

Endothelial cells (50,000 cells per well) were seeded in a 96-well plate in serum dilution buffer (SDB) (1× annexin V buffer (51-66121E, BD Pharmingen), 1 mM MgCl_2_ (68475, Sigma) and 1% BSA (A9576, Sigma)). Pooled normal human serum (NHS, Complement Technology) or cynomolgus monkey serum (NHP01SRM, BioIVT) diluted in SDB were added to appropriate wells at a final concentration of 25% and incubated for 30 min at 37 °C. For negative controls, cells were treated with 25% serum containing 10 mM EDTA (15575038, Thermo Fisher) to inactivate complement. After incubation, cells were washed and stained with phycoerythrin-conjugated anti-C3b antibodies at a 1:100 dilution (846104, BioLegend) and Ghost Dye Red 780 viability dye at a 1:500 dilution (13-0865, Tonbo Biosciences) for 30 min at 4 °C in the dark. Cells were washed twice, immediately acquired on a BD FACSymphony A3 cytometer and analysed in Flow Jo. C3b deposition was plotted as MFI and statistics were calculated using GraphPad Prism v8.2.0.

### Complement-dependent cytotoxicity assay

One day before the assay, endothelial cells (3,000 cells per well) were seeded in a 96-well plate (3595, Corning) in endothelial cell base medium. The next day, adherent cells were washed once with SDB, treated with 25% diluted sera (described above) containing 250 nM Cytotox Red viability dye (4632, Essen Biosciences), immediately placed in an Incucyte SX5 live-cell analysis system (model S3, Sartorius) and incubated at 37 °C in a CO_2_ incubator for the durations indicated in the figure legends. The number of Cytotox Red-positive cells was counted by the Incucyte software, and total cell counts were determined manually from phase contrast images. Complement-dependent cytotoxicity was calculated by normalizing the number of Cytotox Red-positive cells to the total number of cells. Data were plotted as percent Cytotox Red-positive cells, and statistics were calculated using GraphPad Prism v8.2.0.

### aPC assay

The day before the assay, endothelial cells (20,000 cells per well) were seeded in a 48-well plate (FB012930, Thermo Fisher) in endothelial cell base medium. The next day, adherent cells were washed once with assay buffer (10 mM Tris HCl (15567-027, Thermo Fisher), 150 mM NaCl (S5886, Sigma), 5 mM CaCl_2_ (C1016, Sigma), 0.1% BSA (A9576, Sigma), pH 7.5), and then, where applicable, incubated with 40 μg ml^−1^ RCR-252 (2× final concentration; MA5-33375, Thermo Fisher) and/or 4 μg ml^−1^ PBS-01 (2× final concentration; ab6980, Abcam) for 1 h at room temperature in assay buffer. After incubation and without removing the blocking antibodies, cells were treated with 0.1 U ml^−1^ thrombin (605190, Sigma) and 150 nM protein C (539215, Sigma), both diluted in assay buffer, for 60 min at 37 °C. After incubation, 2 U ml^−1^ hirudin (H0393, Sigma) diluted in assay buffer was added to quench thrombin activity and the plate was incubated for 5 min at 37 °C. The solutions from each well were transferred to a 96-well plate, alongside a serial dilution of purified human aPC (HCAPC-0080, Prolytix) diluted in assay buffer to produce a standard curve. Spectrozyme PCa chromogenic substrate (336, Biomedica Diagnostics), diluted to 1 mM in imidazole buffer (0.1985 g ml^−1^ imidazole (I5513, Sigma), 0.03535 g ml^−1^ Tris (BP152, Thermo Fisher), 0.12675 g ml^−1^ NaCl and 250 mM HCl, pH 8.5), was added and absorbance read at 405 nm every 30 s for 15 min on a microplate reader (FilterMax F5, Molecular Devices). Initial velocity of the reaction was calculated (slope of the initial linear part of the curve), and aPC concentrations were determined using the aPC standard curve. Concentration data were plotted, and statistics were calculated using GraphPad Prism v8.2.0.

### Whole-blood TAT complex assay

The day before the assay, endothelial cells (75,000 cells per well) were seeded in a 24-well plate (353226, Corning) in endothelial cell base medium. Fresh whole blood was collected in-house by a phlebotomist (Quadrant Health) in a Vacutainer serum collection tube (367820, BD Biosciences) containing 0.5 U ml^−1^ heparin (H3393, Sigma), 225 μl added to the adherent cells after a wash with 1× PBS and incubated at 37 °C on an Orbitron plate rocker (201100, Boekel Scientific) for 40 min. After incubation, non-clotted blood was transferred to Eppendorf tubes and centrifuged at 1,500*g* for 10 min. For baseline TAT concentration, whole blood was centrifuged at the same time blood was being added to cells. Plasma was collected and frozen on dry ice until use. Plasma samples were used in a TAT complex ELISA (ab108907, Abcam) according to the manufacturer’s instructions with TAT concentrations calculated based on a standard curve of purified human TAT complex, read on an Envision 2105 Multimode Plate Reader (2105-0010, Perkin Elmer). Concentration data were plotted, and statistics were calculated using GraphPad Prism v8.2.0.

### SIRPα reporter assay

The PathHunter Jurkat SIRPα Signaling Bioassay Kit (93-1135Y19, Eurofins DiscoverX) was used to assess human CD47 function. Porcine KECs (30,000 cells per well) were incubated with or without increasing concentrations of an anti-human CD47 blocking antibody (clone B6H12.2) followed by addition of Jurkat SIRPα reporter cells (10,000 cells per well). Cells were incubated at 37 °C in a humidified CO_2_ incubator (5% CO_2_) for 24 h, and kit instructions were followed for signal detection. Luminescence of the plates was read on a microplate reader (FilterMax F5, Molecular Devices) according to the manufacturer’s instructions. Relative luminescence units were plotted and statistics were calculated using GraphPad Prism v8.2.0.

### Caspase 3/7 assay

Endothelial cells (70% confluent in a 10-cm gelatin-coated tissue culture dish) were treated with 50 ng ml^−1^ recombinant human TNF (210-TA-100/CF, R&D Systems) in complete endothelial cell medium overnight. Cells were harvested from the plate and 10,000 cells were added to a 96-well flat-bottom white plate (07200589, Thermo Fisher), and caspase 3/7 activity was determined using the Caspase-Glo 3/7 Assay System (G8093, Promega). In brief, an equal volume of fresh Caspase-Glo 3/7 reagent was added to the wells, the plate incubated for 30 min at room temperature in the dark and then read on the Envision 2105 Multimode Plate Reader (2105-0010, Perkin Elmer). Data were plotted, and statistics were calculated using GraphPad Prism v8.2.0.

### NHP recipients

Male and female cynomolgus monkeys (*Macaca fascicularis*; Bioculture US LLC and Alpha Genesis) weighing 4–12 kg (estimated 3–8 years of age) were used. Monkeys were screened for the presence of anti-porcine IgG and IgM (described above), and animals with low anti-porcine IgG and anti-IgM were selected as recipients. Yucatan pigs weighing 5–27 kg were used as kidney donors. All animal care, surgical procedures and postoperative care of animals were conducted in accordance with NIH Guidelines for the care and use of primates and The Guide for the Care and Use of Laboratory Animals and were approved by Institutional Animal Care and Use Committees at Duke University (protocol A032-20-02, approved 27 February 2020), University of Wisconsin at Madison (protocol G006507, approved 30 September 2021) and the Massachusetts General Hospital (protocol 2017N000216, approved 20 November 2020). All studies followed the 3Rs principles.

### Kidney transplantation

To prepare the cynomolgus monkey for the procedure, a central venous line was inserted through the internal jugular vein 2–7 days before kidney transplantation. Through the midline incision, the kidney xenograft was transplanted intraperitoneally by anastomosing the renal vein and artery to the vena cava and abdominal aorta, respectively. Ureterovesical anastomosis was performed by the Lich–Gregoir technique without placing a ureteral stent. Bilateral native nephrectomy was performed simultaneously in the majority of the recipients with the exception of M8220 and M6521, where one native kidney was left intact at transplant and then removed around POD 20. Postoperatively, the transplanted kidney was monitored by urine output, ultrasound, clinical chemistry and haematology, as well as protocol biopsies. The central venous line was removed by 2–4 weeks once recipient animals had stable kidney function, to avoid the risk of infection. Long-term survival refers to life-supporting function of more than 3 months in the NHP recipient.

### Histopathological analysis

Protocol renal biopsies were obtained every 2–4 months in recipients with stable function as well as when rejection was suspected owing to a rise in creatinine. Tissue was processed for light microscopy. Following euthanasia of a monkey, a complete autopsy was performed for histopathological examination of the renal xenograft, lymph nodes, heart, lung, liver, pancreas, thymus and skin. Xenograft haemotoxin and eosin and periodic acid–Schiff-stained samples were scored by current Banff criteria including C4d deposition^42^.

### Reporting summary

Further information on research design is available in the Nature Portfolio Reporting Summary linked to this article.

## Online content

Any methods, additional references, Nature Portfolio reporting summaries, source data, extended data, supplementary information, acknowledgements, peer review information; details of author contributions and competing interests; and statements of data and code availability are available at 10.1038/s41586-023-06594-4.

### Supplementary information

Supplementary Information This file contains Supplementary Methods, Supplementary Tables 1-6, Supplementary Figures 1-5, and additional references.

Reporting Summary

### Source data

Source Data Fig. 1

Source Data Fig. 2

Source Data Fig. 3

Source Data Fig. 4

Source Data Extended Data Fig. 1

Source Data Extended Data Fig. 2

Source Data Extended Data Fig. 4

Source Data Extended Data Fig. 5

Source Data Extended Data Fig. 6

Source Data Extended Data Fig. 7

Source Data Extended Data Fig. 8

## Figures and Tables

**Extended Data Table 1:**
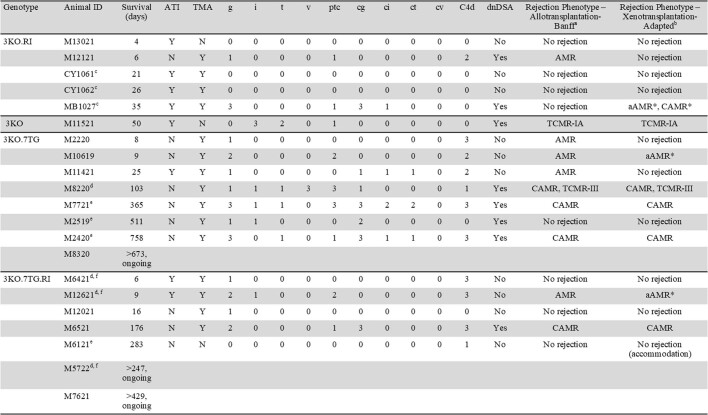
Summary of Banff lesion scoring and pathologic diagnoses at necropsy

>, recipient surviving at the cutoff time of 31 March 2023; 3KO, three knockout (*GGTA1/CMAH/B4GALNT2/B4GALNT2L* knockout); 7TG, seven human transgenes (*THBD*, *PROCR*, *TNFAIP3*, *HMOX1*, *CD46*, *CD55*, and *CD47*); AMR, antibody mediated rejection by Banff Scoring; ATI, acute tubular injury; aAMR, acute AMR in xenotransplantation context; aAMR*, presumed acute AMR in xenotransplantation context; CAMR, chronic active AMR in xenotransplantation context; CAMR*, presumed chronic active AMR in xenotransplantation context; CAMR, chronically active antibody mediated rejection by Banff; C4d, C4 split product; cg, glomerular basement membrane double contours also called transplant glomerulopathy Banff lesion; ci, interstitial fibrosis Banff lesion; CoB, costimulation blockade; ct, tubular atrophy Banff lesion; cv, vascular fibrous intimal thickening Banff lesion; dnDSA, de novo donor specific antibody; g, glomerulitis Banff lesion; I, interstitial inflammation Banff lesion; ptc, peritubular capillaritis Banff lesion; RI, retroviral inactivation; t, tubulitis Banff lesion; TCMR, T cell mediated rejection; TMA, thrombotic microangiopathy; v, intimal arteritis Banff lesion.

^a^Rejection phenotype graded according to most recent Banff criteria from allotransplantation (Roufosse C, *et al.*, 2018).

^b^Rejection phenotype graded according to Supplementary Table 6.

^c^Denotes transplants carried out at additional sites (Duke University for MB1027 and University of Wisconsin for CY1061 and CY1062).

^d^Denotes animals receiving CoB with TNX-1500.

^e^Xenografts from these recipients contained benign cysts.

^f^Denotes animals which did not receive MMF as part of their maintenance immunosuppression.

## Data Availability

All raw and processed sequencing data generated in this study have been submitted to the NCBI Gene Expression Omnibus (https://www.ncbi.nlm.nih.gov/geo/) and the Sequence Read Archive (https://www.ncbi.nlm.nih.gov/sra) under BioProject PRJNA870308. Additional data that support the findings of this study, including IHC data from individual NHP transplants, are available from the corresponding author on reasonable request. Source data are provided with this paper.
