# Supplementary material for: Design and testing of a humanized porcine donor for xenotransplantation
Source: Nature. 2023 Oct 11;622(7982):393–401. doi: 10.1038/s41586-023-06594-4 (PMC10567564; doi:10.1038/s41586-023-06594-4)
Supplement: Supplementary file 1 — This file contains Supplementary Methods, Supplementary Tables 1-6, Supplementary Figures 1-5, and additional references. [file 41586_2023_6594_MOESM1_ESM.docx]

**Supplementary Methods**

**Derivation and culture of ear punch derived cells (EPDCs)**

Ear punch samples (5 mm diameter) were excised from newborn Yucatan pigs, minced, and digested with collagenase type IV (1 mg/mL, dissolved in EPDC culture media described below) (C5138, Sigma Aldrich, St. Louis, MO) at 37°C in a shaker (100 RPM) for 1 hour. The cell suspension was centrifuged at 400 xg for 4 minutes at room temperature, cell pellet resuspended in EPDC culture media, plated onto two 10-cm culture dishes (353003, CORNING, Corning, NY) pre-coated with 0.2% gelatin (J62699, Alfa Aesar, Tewksbury, MA), and cultured as described below. Porcine primary EPDCs were cultured in Dulbecco’s modified Eagle’s medium (DMEM) (10569010, Thermo Fisher), supplemented with 20% heat-inactivated fetal bovine serum (FBS) (10438026, Thermo Fisher), 10 mM HEPES (15630080, Thermo Fisher), and 100 U/mL Penicillin/Streptomycin (Pen/Strep) (15140122, Thermo Fisher). Immediately before use, recombinant human fibroblast growth factor, basic, (bFGF) (PHG0266, Thermo Fisher) was added to a final concentration of 5 ng/mL. All cell culture was conducted in a humidified tri-gas (5% CO_2_, 90% N_2_, and 5% O_2_) incubator (Heracell VIOS 160i, 51033557, Thermo Fisher) set at 37°C.

**Derivation and culture of primary porcine kidney endothelial cells (KECs)**

Kidney cortex (5 cm^2^) was excised, minced, and digested in collagenase type IV (1 mg/mL) dissolved in HBSS (MT-20023CV, Thermo Fisher) for 30 minutes at 37^o^C. The cell suspension was filtered, the pellet was resuspended in endothelial cell base medium of DMEM (10313021, Thermo Fisher), 10% heat-inactivated FBS, 1X GlutaMAX (35050061, Thermo Fisher), 10 mM HEPES, 100 U/mL Pen/Strep, and 0.5 mg/mL Primocin (ant-pm-1, InvivoGen, San Diego, CA), and cell suspension plated into 1-3 T-175 flasks pre-coated with 0.2% gelatin solution. After plating the cells, 100 µg/mL (2X) endothelial cell growth supplement (ECGS) (354006, Corning) was added to each flask for culturing. The cells were stained with Alexa Fluor 647-conjugated porcine CD31 antibody (FAB33871R-100UG, R&D Systems, Minneapolis, MN), and sorted for CD31 expression on a Beckman Coulter MoFlo Astrios EQ, BD FACSMelody, or Thermo Fisher Bigfoot Spectral cell sorter. Cell culture was conducted in a humidified incubator set at 37°C in 5% CO2.

**Derivation of primary aortic endothelial cells (AECs)**

A segment of the thoracic and abdominal aorta was excised, cut open lengthwise, submerged in a conical tube containing 20 mL of HBSS, and digested with collagenase type IV (0.5 mg/mL) on a rotating platform at room temperature for 1 hour. Subsequently, endothelial cells were scraped from the inner lining of the aorta and combined with the dissociation solution. The sample was filtered, centrifuged, and the pellet was resuspended in endothelial medium supplemented with 100 µg/mL (2X) ECGS, and plated directly on a gelatin coated 10-cm tissue culture dish. Cell sorting for porcine CD31 and post-sort culturing was performed as described above for KECs. Cell culture was conducted in a humidified incubator set at 37°C in 5% CO2.

**Derivation and culture of kidney cortex derived cells (KCDCs)**

Tissue processing was performed as described above for KECs except cells (without sorting) were plated in T-175 flasks in a complete kidney cell media of DMEM (10313021, Thermo Fisher), 20% heat-inactivated FBS, 100 U/mL Pen/Strep, 1x non-essential amino acids (NEAA) (11140-050, Gibco), 0.1% beta Mercaptoethanol (ES-007-E, EMD Millipore) without the addition of ECGS. KCDC culturing was conducted in a humidified tri-gas incubator set at 37°C.

**Generation of individual gene and transcription cassette-expressing kidney cells**

For each plasmid, complementary DNA for a gene or a polycistronic cassette carrying 2-3 genes was separated from a marker GFP sequence by an internal ribosome entry site (IRES) sequence, expression driven by the CAG promoter, and terminated by the bovine growth hormone polyadenylation signal. Constructs were assembled using the HiFi DNA Assembly Master Mix (E2621, NEB, Ispwich, MA) per manufacturer’s instructions. Transgene expression plasmid was electroporated into porcine cells with the Neon^TM^ Transfection System (MPK5000, Thermo Fisher) according to manufacturer’s instructions, with 500 ng transgene plasmid DNA and 25 ng Super *PiggyBac transposase* vector (PB210PA-1, SBI, Palo Alto, CA) in Opti-MEM medium (31985-062, Gibco). Transfected cells were selected through two rounds of sorting for GFP expression on a Beckman Coulter MoFlo Astrios EQ or Thermo Fisher Bigfoot Spectral cell sorter. The *CD46-CD55-CD47* and *PROCR-THBD* cassettes were expressed from the KECs, while those for the *TNFAIP3-HMOX1* cassette from the KCDCs.

**Isolation of RNA from porcine kidney samples and CD31+ and CD31- kidney endothelial cells**

Snap-frozen tissue collected from the contralateral kidney, biopsy or necropsy samples was pulverized using a pre-chilled BioPulverizer (59012MS, BioSpec). Total RNA was extracted using homogenizing columns (79656, Qiagen), DNase digestion (79256, Qiagen) and the RNeasy kit (74106, Qiagen) according to manufacturer’s instructions. Primary cells (0.75x10^6^ - 1.5x10^6^ cells) were pelleted and lysed directly in 350 µL Buffer RLT Plus (1053393, Qiagen). Each sample was passed through a QIAshredder (79656, Qiagen) by centrifuging at 18,000 xg for 2 minutes. Total RNA was isolated from each homogenized lysate using the RNeasy Plus Mini Kit (74134, Qiagen) following manufacturer’s protocol. Purified RNA was eluted in 30-50 µL RNAse-free water.

**Transgene analysis by flow cytometry on endothelial cells**

Porcine KECs, AECs, HUVECs (PCS-100-010, ATCC, Manassas, VA), and HGMVECs (ACBRI 128, Cell Systems, Kirkland, WA) were stained with anti-human CD46 (352409, BioLegend, San Diego, CA), anti-human CD55 (555694, BD Biosciences, San Jose, CA), anti-human CD47 (11-0479-41, Thermo Fisher), anti-human TM (740604, BD Biosciences), or anti-human EPCR (557950, BD Biosciences) antibodies for 30 minutes in the dark. Afterwards, the cells were washed twice with 1X PBS and immediately acquired on a BD FACSymphony A3 cytometer and analyzed using Flow Jo software (Ashland, OR). Gating strategy is provided in Supplementary Fig. 2.

**Western blot analysis of human TNFAIP3 and HMOX1 proteins**

Frozen cell pellets were processed according to standard protocols. Membranes were blocked, incubated in primary antibody (anti-TNFAIP3, ab92324, abcam) for 1.5 hours at room temperature (RT) in 5% BSA in Phosphate Buffer Saline with 0.1% Tween (PBS-T) at a 1:500 dilution and probed with a goat anti-rabbit IgG secondary antibody (ab216773, abcam) at 1:5000 dilution. An image was taken on a LI-COR Odyssey (Lincoln, Nebraska). Beta actin was detected with a primary antibody against all known isoforms of actin (BDB612656, BD Biosciences) and a goat anti-mouse IgG secondary (ab216776, abcam) and used as a loading control.

**Human CD47-Fc binding to human or cynomolgus monocytes**

Human monocytes were enriched from PBMCs by magnetic separation (130-117-337, Miltenyi Biotech). Human or cynomolgus monkey monocytes (IQB-MN1-CD14-2, IQ Biosciences, Berkley, CA) (100,000 cells/well) were plated in a 96-well round bottom tissue culture plate (FB012932, Thermo Fisher), treated with 1X PBS alone or 20 µg/mL recombinant human CD47-Fc chimera protein (4670-CD-050, R&D Systems) in 1X PBS, and incubated at 37°C in humidified CO2 incubator (5% CO_2_) for 60 minutes. The unbound CD47-Fc protein was washed out and the bound CD47-Fc protein detected with a PE-conjugated anti-human IgG Fc antibody (409304, R&D Systems), fixed, and acquired on a FACSymphony A3 Cell Analyzer. The Non-Human Primate (NHP) and human monocytes were stained with a PE-Cy7 conjugated anti-human SIRPα antibody (323807, BioLegend) to measure SIRPα surface expression. Data was plotted and statistics were calculated using GraphPad Prism, v8.2.0.

**Fluid challenge analysis**

After completion of the mGFR study (described above), the same group of four-month-old wild-type and the 3KO.7TG Yucatan swine were used in a series of fluid challenges. First the animals were water fasted (fluid restriction) for 22 hours. After 24 hours recovery with intermittent access to water, 0.9% saline (20 mL/kg) was dosed as a hydration challenge. Finally, six hours later, furosemide (1 mg/kg) was administered. Body weights, serum chemistry and hematology were assessed before and after the fluid restriction as well as before the saline dose, six hours after the saline dose and six hours after the furosemide dose. Urine was collected for a two-hour baseline prior to study start, and then in six-hour increments throughout the fluid restriction phase. Baseline urine was again collected before the saline challenge, and then throughout the remainder of the study in one-hour bins immediately after saline or furosemide dose and then in two 2.5 hour bins for a total of six hours. Urine volume was measured for each time and urinalysis and urine chemistry performed.

**Custom Payload 15S genome build**

For alignment of Nanopore whole genome sequencing (WGS) and direct RNA-seq reads (dRNA-seq), a modified Ensembl Sscrofa11.1 genome was constructed with a custom script that inserts the validated Payload 15S sequence flanked by the *loxP* and *lox2272* sites at chr6: 111497713 (minus strand) (chr6: 59,345,875 plus strand) (*PPP1R12C* or *AAVS1 site*).

**Nanopore whole-genome sequencing**

WGS libraries were constructed using LSK109 kit (Oxford Nanopore) according to manufacturer’s protocol and without modification and libraries sequenced using R9.4.1 PromethION flowcell chemistry. Nanopore reads were aligned to the custom Payload 15S genome build, using winnowmap2 v2.03 with default parameters and k-mer size 15^52^. BAM files were subsetted for full length reads spanning both junctions and sorted using samtools v1.7 ^51^ and visualized using Integrative Genomics Viewer (IGV version 2.12.3)^53^.

**Nanopore direct RNA-seq**

Total RNA was extracted from 20-30 mg snap-frozen kidney tissue using the AllPrep DNA/RNA Micro Kit (80284, Qiagen) as per the manufacturer’s protocol. Libraries were prepared from 5 µg total RNA using the Nanopore Direct RNA Sequencing Kit (SQK-RNA002, Oxford Nanopore) as per the manufacturer’s protocol with the optional reverse transcription step. Libraries were sequenced on a Nanopore GridION instrument using R9.4.1 flow cell chemistry. FASTQ files were mapped to the custom Payload 15S genome build with minimap2 (2.22-r1101)^54^ and a custom script was used to order the mapped reads for visualization in IGV (https://github.com/egenesis/nanopore_igv_issue_fix_sy.git)^55^.

**Illumina RNA-seq and computational analysis**

Libraries were generated using the Illumina Stranded mRNA kit (20040534, Illumina) on 500 ng of total RNA. Library concentrations were quantified using the Qubit 3 (Q33216, Thermo Fisher) high sensitivity dsDNA assay (Q33231, Thermo Fisher), diluted to a final loading concentration of 650 pM, and sequenced on an Illumina NextSeq 2000 using a P3 100 cycle reagent kit (20040559, Illumina). Transcript sequences were generated with gffread (0.12.7) ^56^ using a modified ENSEMBL Sscrofa11.1 FASTA assembly and v105 GTF annotation with unplaced contigs/scaffolds removed, and PL15S FASTA sequence and GTF annotation added as needed as a separate contig. For samples derived from transplanted organs, the ENSEMBL *Macaca fascicularis* 6.0 FASTA assembly and v105 GTF annotations with unplaced contigs/scaffolds removed were appended to the pig reference. Transcripts were indexed using salmon index (1.6.0)^57^ with standard settings, and using the whole genome as a decoy, and RNA-seq read counts were quantified at the transcript level with salmon quant (mapping-based mode) with custom settings: 100 bootstrap replicates and GC content and sequence bias correction were applied. Transcript abundances were aggregated to gene level using tximport (1.22.0)^58^, only protein coding and lncRNA genes were retained, and normalized rlog counts were generated with DESeq2 (1.34.0)^59^ using sample collection source (i.e. contralateral, biopsy, and necropsy) and Pig ID as an unblinded covariates for the kidney data, and sample type (i.e., CD31-, CD31+ KEC) and Pig ID for the KEC data. For each PL15S cassette the mean rlog value for all genes in the cassette was computed before plotting with ggplot2. The ComplexHeatmap (2.10.0)^60^ library was used for heatmap creation. Code for payload expression (<https://github.com/egenesis/Nature_2023_RNA-seq>) and KEC identity ([https://github.com/egenesis/Nature_2023_scRNAseq)](https://github.com/egenesis/Nature_2023_scRNAseq) are available on github.

**scRNA-seq of dissociated kidney cells and computational analysis of 3’ scRNA-seq**

Kidney samples were dissociated into single cells as described above and cells processed for scRNA-seq. The cells were resuspended in RBC lysis buffer (11814389001, Roche, Basel, Switzerland) for 2 minutes on ice. Lysis was terminated by adding 1X HBSS with 20% FBS, the cells were centrifuged and the pellets resuspended in 1X HBSS and filtered through a 70-µm filter. Cells were manually counted by trypan blue exclusion, and dead cell removal was performed using a Dead Cell Removal Kit (130-090-101, Miltenyi Biotec) following manufacturer’s instructions. After dead cell removal, cells were recounted and resuspended in complete medium at a concentration of 600-1200 cells/μL. Cell suspensions were used to generate scRNA-seq libraries, using 10X Genomics Chromium Next GEM Single Cell 3’ v3.1 (Dual Index) (1000121, 10X Genomics, Pleasanton, CA), per manufacturer’s instructions. The assembly and annotation previously generated for RNA-seq was indexed using STARsolo (2.7.9a)^61^ –genomeGenerate with standard settings. 10X 3’ scRNA-seq was quantified using STARsolo with additional settings –soloCellFilter EmptyDrops_CR, --soloMultiMappers EM, --clipAdapterType CellRanger4, --outFilterScoreMin 30, --soloCBmatchWLtype 1MM_multi_Nbase_pseudocounts, --soloType Gene, --soloUMIdedup 1MM_CR, --soloUMIfiltering MultiGeneUMI_CR, and –soloUMIlen 12. Counts were loaded into R (4.1.2) using DropletUtils (1.14.2)^62^ and cells filtered based on number of unique molecular identifiers (UMIs) and detected features, percentage of mitochondrial UMIs, percentage of protein-coding and lncRNA UMIs, complexity (number of detected features versus total UMIs per cell), and doublet removal using scDblFinder (1.8.0)^63^. Counts were normalized using scran quickCluster, computeSumFactors, and logNormCounts per scran (1.22.1) guidelines^64^. Cell-cycle was assigned using Seurat (4.1.0)^65^ CellCycleScoring. Batch effects were removed using scVI (0.15.5)^66^ with mitochondrial UMI percentage, Seurat S and G2M phase cell cycle scores, porcine donor, and kidney region (i.e. cortex or medulla) as covariates. Cells were clustered on the 20 latent dimensions from scVI using the bluster (1.4.0) (<https://www.bioconductor.org/packages/release/bioc/html/bluster.html>) louvain algorithm on shared nearest neighbors (k=3) via scran clusterCells. UMAP manifold was created via scater (1.22.0)^67^ runUMAP with 20 nearest neighbors and a minimum distance of 0.2. Cluster marker genes were computed using scran scoreMarkers and cell types assigned using known markers^68^ and the molecular signature database^69^ cell type signature enrichment using the fgsea (1.20.0)^70^ fora function on the top 50 features per cluster by minimum Cohen’s d log fold change. UMAP and jitter plots were generated using either the scater ggcells function, or scuttle (1.6.2) makePerCellDF in addition to ggplot2 (3.2.0). Code for scRNA-seq analysis is available on github (<https://github.com/egenesis/Nature_2023_scRNAseq>).

**Amplicon sequencing for 3KO and PERV KO genotyping**

Genomic DNA samples were quantified using Qubit 2.0 Fluorometer (Invitrogen, Carlsbad, CA). A series of primers were designed against the region of interest and synthesized through Twist Biosciences (South San Francisco, CA). After amplification, amplicons were pooled at equal molar concentration. Sequencing libraries were prepared using NEBNext Ultra II DNA Library Prep Kit according to manufacturer’s instructions. The adaptor-ligated sequencing libraries were validated on the Agilent TapeStation (Agilent Technologies, Palo Alto, CA), and quantified by Qubit 2.0 Fluorometer (Invitrogen, Carlsbad, CA) as well as by quantitative PCR (KAPA Biosystems, Wilmington, MA). DNA libraries were multiplexed in equal molar mass and loaded on an Illumina MiSeq or NextSeq 2000 instrument according to manufacturer’s instructions (Illumina, San Diego, CA). The sequencing in the MiSeq was performed using a 2x250 paired-end (PE) configuration; image analysis and base calling are conducted by the MiSeq Control Software on the instrument. The sequencing in the NextSeq 2000 was performed using a 2x150 PE configuration; image analysis and base calling are conducted by Illumina Dragen basecalling software version 3.7.4 on a Nextseq 2000.). Illumina Reagent/kits for DNA library sequencing cluster generation and sequencing were used for enriched DNA sequencing.

The quality of the sequencing data was examined using FastQC (0.11.9) (http://www.bioinformatics.babraham.ac.uk/projects/fastqc/). Aadapters were trimmed using fastp (0.20.0) (<https://doi.org/10.1093/bioinformatics/bty560>), and reads with average quality lower than Q20 were removed. Primers were then trimmed using Cutadapt (3.7) (https://doi.org/10.14806/ej.17.1.200), and reads with no primers were removed. The preprocessed data were then aligned to the reference sequences and the edit was quantified using Crispresso2^71^. Modified reads are defined as reads with indels within a 20bp window centered around the predicted cleavage site for 3KO or between cleavage sites for PERV KO. Genotypes supported by less than 0.5% of the reads were considered background noise and ignored. The percentage of modified reads and unmodified reads were reported by the software in the output file “CRISPResso_quantification_of_editing_frequency.txt” and plotted using ggplot2 (3.2.0). For 3KO, alleles around the cleavage site were aggregated and plotted using a custom python script (revised based on the python script from <https://github.com/pinellolab/CRISPResso2/blob/master/scripts/plotCustomAllelePlot.py>). Alleles were examined to confirm all copies of the gene were edited. Alleles supported by less than 1% of the reads were ignored. The percentage of reads supported each genotype/indel size were then plotted using the output data from the script.

**Animal housing, husbandry and pain management**

All housing, husbandry and pain management practices for swine and NHP follow recommendations outlined in the Guide for the Care and Use of Agricultural Animals in Research and Teaching and the Guide for the Care and Use of Laboratory Animals. Specifically, piglets and NHPs are group housed where possible with visibility and cage-side contact with other animals in situations where animals cannot be group housed. Pig and NHP cages are cleaned as often as necessary to provide clean, dry spaces and to mitigate health concerns. In general pigs and NHP are observed at least once daily but more frequently if health concerns are noted, after surgical procedures and for newborn pigs that require more attention. Pre-wean and nursery piglets are fed ad libitum. Older pigs are limit-fed based on weight, and NHP are fed twice daily. All animals receive water ad libitum. Potential pain in pigs is managed with intramuscular flunixin (Banamine) prior to procedures and twenty-four hours later. Any lingering discomfort is monitored daily and treated on an as needed basis based on veterinary staff recommendations. In NHP, subcutaneous or intramuscular buprenorphine and/or hydromorphone and meloxicam is administered for up to two days following surgery. Intravenous fentanyl, ketamine and lidocaine may also be infused during procedures. Local anesthesia (Bupivacaine) is also infiltrated around surgical wounds at the time of skin closure.

**Immune suppression regimen**

The recipients were treated with anti-CD20 mAb (20 mg/kg, NIH NHPRR, Boston, MA) before transplant. Some recipients received additional doses at approximately 60-day intervals until POD 180 to ensure B-cell depletion. Rabbit polyclonal anti-rhesus thymocyte globulin (5 mg/kg, rhATG, AB_2716327, NIH NHPRR, Boston, MA) was given on days -1 and 0 as additional induction therapy. During the transplant procedure, two doses of anti-CD154 mAb (25 mg/kg, 5C8H1, AB_2716324, NIH NHPRR, or TNX-1500, Tonix Pharmaceuticals, Chatham, NJ) were administered. Additional 20 mg/kg doses were given on days 2, 5, 7, 12, and then weekly for the course of the study. To prevent possible thrombotic complications from the anti-CD154 mAb 5C8H1, 1 mg/kg of ketorolac (Pfizer, New York, NY) was administered prior to each dose with 5C8H1. Daily mycophenolate mofetil (200 mg, Genentech, San Francisco, CA) was given orally. During the first 2 months, tacrolimus (Astellas, Northbrook, IL) was administered by intramuscular injection to maintain trough levels 5-10 ng/ml. Daily solumedrol (40 mg, Pfizer, New York, NY) was tapered beginning at 2 weeks with dosing ending by day 30. Weekly or daily subcutaneous Epogen (Amgen, Thousand Oaks, CA) injections were given to maintain hematocrit at 30% or above. In response to weight loss or infection, maintenance immunosuppression was occasionally modified by discontinuing MMF or increasing the time between anti-CD154 doses from weekly to every 10 days.

**Lymphocyte subset analysis**

Peripheral blood cells from red blood cell-lysed whole blood were labelled with a combination of the following antibodies: anti-human CD3 (SP34-2), anti-human CD4 (L200), anti-human CD8 (SK1), anti-human CD45RA (5H9) (BD Pharmingen), and anti-human CD20 (2H7) (BioLegend). Stained samples were acquired and analyzed using FACS Verse (BD Biosciences) and FlowJo. Gating strategy is provided in Supplementary Fig. 4.

**Supplementary Table 1.**

Molecular incompatibilities exist among pigs, humans, and old world monkeys for cell surface proteins carried in Payload 15S

| **H. sapiens** | | | | **S. scrofa** | | **M. fascicularis (cyno)** | | **P. anubis (baboon)** | | **M. mulatta (rhesus)** | |
| --- | --- | --- | --- | --- | --- | --- | --- | --- | --- | --- | --- |
| **ENSEMBL Gene ID** | **Gene Name** | **Alias** | **UniProt/**  **SwissProt Protein ID** | **UniProt/**  **SwissProt Protein ID** | **Identity (%) to H. sapiens** | **UniProt/**  **SwissProt Protein ID** | **Identity (%) to H. sapiens** | **UniProt/**  **SwissProt**  **Protein ID** | **Identity (%) to H. sapiens** | **UniProt/**  **SwissProt Protein ID** | **Identity (%) to H. sapiens** |
| ENSG00000117335 | *CD46 ​* | MCP ​ | P15529 | O02839 | 40.46​ | A0A2K5WCS2 | 77.44 ​ | A0A096NAP4 | 85.71​ | F7DTG0 | 77.69​ |
| ENSG00000196352 | *CD55 ​* | DAF ​ | P08174 | Q9GLM2 | 36.77​ | A0A2K5X1U9 | 78.77 ​ | A0A2I3LDB2 | 68.47​ | A0A1D5R106 | 78.77​ |
| ENSG00000196776 | *CD47*​ |  | Q08722 | Q9GKE8 | 68.42​ | A0A2K5X4I2 | 80.10 ​ | A0A096NQZ6 | 98.45​ | A0A5F8AK49 | 98.76​ |
| ENSG00000101000﻿ | *PROCR ​* | EPCR ​ | Q9UNN8 | C6EQ34 | 69.42​ | A0A2K5W826 | 93.7 ​ | A0A096NUQ2 | 93.7​ | A0A5F7ZJY1 | 67.17​ |
| ENSG00000178726 | *THBD* ​ | TM ​ | P07204 | A0A4X1V0Q9 | 68.84​ | G7PH40 | 83.3​ | ENSPANT00000063726* | 81.3​ | G7N337 | 87.83​ |

**Supplementary Table 2.**

CRISPR guide RNA sequences were used to edit the porcine genome

| **Genomic Locus/Gene** | **sgRNA Name** | **Sequence (5’ to 3’)** |
| --- | --- | --- |
| *AAVS1* | sgRNA 1 | GGCCCAGAACCAGAAAGAGG |
| *GGTA1* | sgRNA 4 | GCTGCTTGTCTCAACTGTAA |
| *CMAH* | sgRNA 2 | GAAGCTGCCAATCTCAAGGA |
| *B4GALNT2* | sgRNA 15 | AGCTCGAACACTTTCAGAGG |
| PERV  Reverse Transcriptase | sgRNA 3N | TCTGGCGGGAGCCACCAAAC |
|  | sgRNA 5N | GGCTTCGTCAAAGATGGTCG |
|  | sgRNA 9N | TTCTAAGCAGTCCTGTTTGG |

**Supplementary Table 3.**

Protein variants used in Payload 15S

| **Gene** | **mRNA** | **Protein** | **Size (aa)** | **Protein Variant** |
| --- | --- | --- | --- | --- |
| *CD46i** | NM_002389.4 | NP_002380.3 | 392 | ABC1 |
| *CD55* | NM_000574.4 | NP_000565.1 | 381 |  |
| *CD47* | NM_001777.4 | NP_001768.1 | 323 | Form 1 |
| *THBD* | NM_000361.2 | NP_000352.1 | 575 |  |
| *PROCR* | NM_006404.5 | NP_006395 | 238 |  |
| *TNFAIP3* | NM_001270508.1 | NP_001257437.1 | 790 |  |
| *HMOX1* | NM_002133.2 | NP_002124.1 | 288 |  |

*This CD46 sequence carried 19 SNPs, incorporated to eliminate aberrant splicing observed in porcine cells and to balance the AT/GC content.

**Supplementary Table 4.**

PCR primers were used in a polymerase chain reaction to genotype gene edited cells

|  | **Primers** | **Forward (-FAM)** | **Reverse** | **Amplicon Length (bps)** |
| --- | --- | --- | --- | --- |
| **IDAA** | *B4GALNT2*_FA | ACCTTGTCTTATTTTAGGCTGT | CACCCTCGGGAATGAGTA | 224 |
|  | *CMAH*_FA | AGGGAGGGCTTTCAAAC | TCAGGCGGCTCTTATTCT | 249 |
|  | *GGTA1*_FA | CGCTCGTTGACTATTCATC | ACTAGGAGATTAGAGGAGAC | 269 |
|  | PERV FA | CGACTGCCCCAAGGGTTCAA | TCTCTCCTGCAAATCTGGGCC | 236 |
| **Conventional**  **PCR** | *AAVS1*_LP KI_5' Junction | CCTGTAGACTCCACTTCC | GATAACTTCGTATAATGTATGC | 1602 |
|  | *AAVS1*_LP KI_3' Junction | GTAGTATATTCCAGAGTGAAGG | CCCAACTGAATGCCTATG | 1347 |
| **Amplicon Sequencing** | *B4GALNT2*_AmpSeq | TTCCCAATCTGTGATCTTTGA | CACCCTCGGGAATGAGTA | 244 |
|  | *CMAH*_AmpSeq | GAGGGAGGGCTTTCAAAC | TCAGGCGGCTCTTATTCT | 250 |
|  | *GGTA1*_AmpSeq | CGCTCGTTGACTATTCATC | ACTAGGAGATTAGAGGAGACT | 269 |
|  | PERV_AmpSeq_Long | GCCTGAGATTACACCCCACTAGC | CCGCAAACTGTACCCCAAGTATG | 353 |
|  | PERV_AmpSeq_Short | CGACTGCCCCAAGGGTTCAA | AGCCTAGGTCAGACAATTCCAGCA | 196 |

**Supplementary Table 5.** Antibodies and reagents were used in immunochemistry analysis

| **Antibody/Reagent** | **Vendor** | **Catalogue Number** |
| --- | --- | --- |
| EPCR, Ms Ab | abcam | ab236517 |
| THBD, Ms Ab | abcam | ab6980 |
| TNFAIP3, Ms Ab | ThermoFisher | MA5-16164 |
| HMOX1, Rb Ab | abcam | ab52947 |
| CD46, Rb Ab | abcam | ab108307 |
| CD55, Rb Ab | abcam | ab133684 |
| CD47, Rb Ab | abcam | ab226837 |
| C5b9 | abcam | ab55811 |
| Goat-anti-mouse-HRP | DAKO | K4001 |
| Goat-anti-rabbit-HRP | DAKO | K4003 |
| Xylene | EMD Millipore | 1330-20-7 |
| FLEX80 | Fisher Scientific | 8315 |
| FLEX95 | Fisher Scientific | 8215 |
| FLEX100 | Fisher Scientific | 8115 |
| TBS | Sigma | T5912-1L |
| TBS-T | ThermoFisher | TA-999-TT |
| Goat serum | EMD Millipore | 566380-10ML |
| Peroxidazed 1 | Biocare Medical | PX968MM |
| Hoechst 33258 | PromoKine | PK-CA707Go-40044 |
| Isolectin B4-FITC | Enzo | ALX-650-001F-MC05 |
| DBA-biotin | Vector Labs | B-1035 |
| Chicken anti-Neu5GC | BioLegend | 146903 |
| Goat anti-Chicken Alexa Fluor 647 | ThermoFisher | A21449 |
| Streptavidin, Alexa Fluor568 | ThermoFisher | S11226 |
| EDTA buffer | Thermo Scientific | AP-9004-500 |
| Citrate buffer | Thermo Scientific | AP-9003-500 |
| ProLong Glass Antifade Mountant | Invitrogen | P36984 |
| Cy-5 Reagent pack | Akoya Biosciences | TF-00103 |
| OPAL Polymer HRP Ms+ Rb | Akoya Biosciences | ARH1001EA |
| OPAL 520 | Akoya Biosciences | FP1487001KT |
| OPAL 690 | Akoya Biosciences | FP1497001KT |

**Supplementary Table 6:** Rejection Phenotyping in Xenotransplantation Context

|  | aAMR | aAMR* | cAMR | cAMR* | ACC | NoR | aTMA | cTMA |
| --- | --- | --- | --- | --- | --- | --- | --- | --- |
| mvi (g+ptc) | >2 | >2 | >2 | >2 | $\leq$2 | $\leq$2 | $\leq$2 | $\leq$2 |
| C4d | + | +/- | + | +/- | + | - | +/- | - |
| cg | 0 | 0 | >0 | >0 | 0 | 0 | 0 | >0 |
| dnDSA | + | +/- | + | + | +/- | - | - | - |
| thrombi | +/- | +/- | +/- | +/- | - | - | + | + |
| v | +/- | +/- | - | - | - | - | - | - |
| Acute AMR (aAMR)  In the context of xenotransplantation, aAMR is defined as the presence of significant microvascular inflammation (mvi>2), C4d deposition, and the presence of de novo DSA. | | | | | | | | |
|  | Because mvi and C4d indicate activity, the following are also presumed to be AMR (aAMR*):   - mvi>2 and positive C4d, even if dnDSA is not detected - mvi>2 and presence of dnDSA, even if C4d is negative | | | | | | | |
| Chronic active AMR (cAMR)  In the context of xenotransplantation, cAMR is defined as the presence of significant microvascular inflammation (mvi>2), transplant glomerulopathy (cg>0), C4d deposition, and the presence of de novo DSA. | | | | | | | | |
|  | Because mvi and C4d indicate activity, while cg indicates chronicity in cAMR, the following are presumed to be cAMR (cAMR*):   - mvi>2, cg>0, and positive C4d, even if dnDSA is not detected | | | | | | | |
| Abbreviations: +, detected; -, not detected; +/-, detected or not detected; ACC, accommodation; aAMR, acute antibody mediated rejection; aAMR*, presumed acute antibody mediated rejection; cAMR, chronic active antibody mediated rejection; cAMR*, presumed chronic active antibody mediated rejection; C4d, complement component C4d (split product of C4 activation); cg, glomerular basement membrane double contours also called transplant glomerulopathy Banff lesion; dnDSA, de novo donor specific antibody (IgG or IgM); g, glomerulitis Banff lesion; mvi, microvascular inflammation which is the sum of glomerulitis and peritubular capillaritis lesion scores; NoR, no rejection; ptc, peritubular capillaritis Banff lesion; aTMA, acute thrombotic microangiopathy; cTMA, chronic thrombotic microangiopathy; v, intimal arteritis Banff lesion. | | | | | | | | |

Discusssion:

TMA is an accepted, but not necessary, feature of aAMR in Banff classification^42^. In the xenografts reported here, TMA was commonly seen at necropsy (15/18). However, TMA occurred in the absence of detectable DSA (8/15) or C4d deposition (6/15), two standard AMR features. Since coagulopathy in this setting could be related to clotting factor incompatibilities between porcine and HP^72,73^, TMA is not included as an indication of AMR. Since CD46 and CD55 block complement activation at C3, the transgenic proteins will not prevent C4d deposition^74^. Thus, C4d deposition may not indicate full complement activation. It is known that C4d deposition commonly occurs in ABO incompatible (ABOi) human renal allografts without evidence of active AMR^75^,and that C4d deposition does not trigger endothelial transcript elevation in human renal allografts with CAMR or ABOi grafts^76^. Therefore, C4d deposition cannot be taken as a reliable indicator of AMR (similar to human ABOi grafts), but as a marker of xenoantibody reacting to the donor endothelium. Of the 11 C4d+ cases, 5 had detectable DSA and 6 did not, suggesting that some anti-endothelial antibodies may be below the limit of detection by the assays used.

The main pathologic feature used in Banff for activity in AMR is microvascular inflammation (peritubular capillaritis (ptc) and glomerulitis (g))^42^. These are commonly added (mi) with a threshold for positivity of 2. Bulk RNA analysis has shown peritubular capillaritis is highly correlated with endothelial transcript elevation in human renal allografts with CAMR^76^. Furthermore, peritubular capillaritis is not a feature of TMA in native kidneys. We have used Banff mi score>2 as a criterion for active AMR. Of the 7 xenokidneys with mi>2, 4 had both C4d and DSA, 2 had C4d without DSA and one had DSA without C4d. None lacked both DSA and C4d, arguing that this criterion requires the presence of either DSA or C4d. Transplant glomerulopathy (cg), or duplication of the glomerular basement membrane, is a nonspecific feature that indicates chronic endothelial injury and can thus be present in chronic TMA or chronic AMR. In the presence of microvascular inflammation, C4d and DSA, this pathologic feature is attributed to chronic, active antibody mediated rejection.


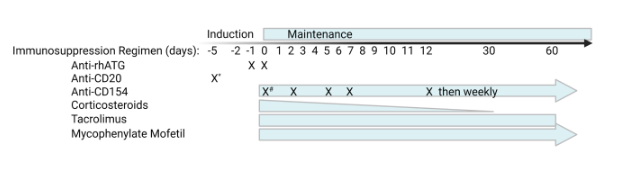


**Supplementary Fig. 1**. **Induction and maintenance immunosuppression regimen used in the NHP transplantation study.** Rabbit anti-rhesus thymocyte globulin (rhATG) and anti-CD20 monoclonal antibody (mAb) were used as induction immunosuppression. After transplantation, anti-CD154 mAb (5C8) or Fc-modified anti-CD154 mAb (TNX-1500) on days 0, 2, 5, 7, and 12, and then weekly (25 mg/kg on day 0 then 20 mg/kg) thereafter, corticosteroids (for 1 month), tacrolimus (for 2 months), and daily mycophenolate mofetil were administered. * indicates that some recipients received additional doses of anti-CD20 at approximately 60-day intervals until POD 180 to ensure B cell depletion. # indicates administration of anti-CD154 before and after xenograft perfusion.

**Supplementary Fig. 2** **Gating strategy for cell sorting and flow cytometry analysis.** **a,** Sorting scheme to enrich for CD31+ endothelial cells. Aortic endothelial cells (AECs) proceeded through just one sort, while kidney endothelial cells (KECs) proceeded through two sorts to establish stable CD31+ purity to be used in assays. **b,** Master gating scheme representative of analysis of cell lines in Fig. 1b, 1c, Extended Data Fig. 6 and 8. **c,** Master gating scheme representative of analysis of cell lines in Fig. 1d, 1e, 3a, 3b, 3d, 3e, Extended Data Fig. 3a, 3c, 4a, 4d, 4f, 5a, and 5b.

| a. | b. |
| --- | --- |
| 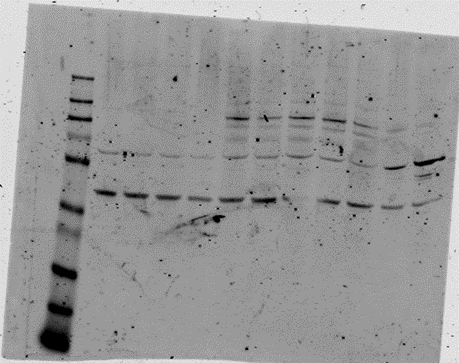 | 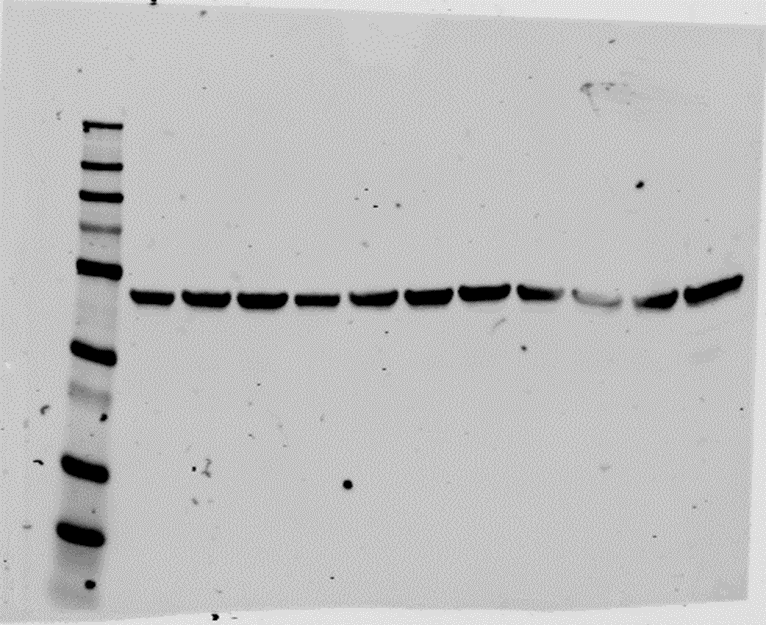 |

**Supplementary Fig 3.** **Uncropped gel images supporting Extended Data Figure 5c.** a. Blot probed with an antibody detecting TNFAIP3 (ab92324, abcam). b. Blot probed with an antibody detecting β-actin as a loading control (BDB612656, BD Biosciences).


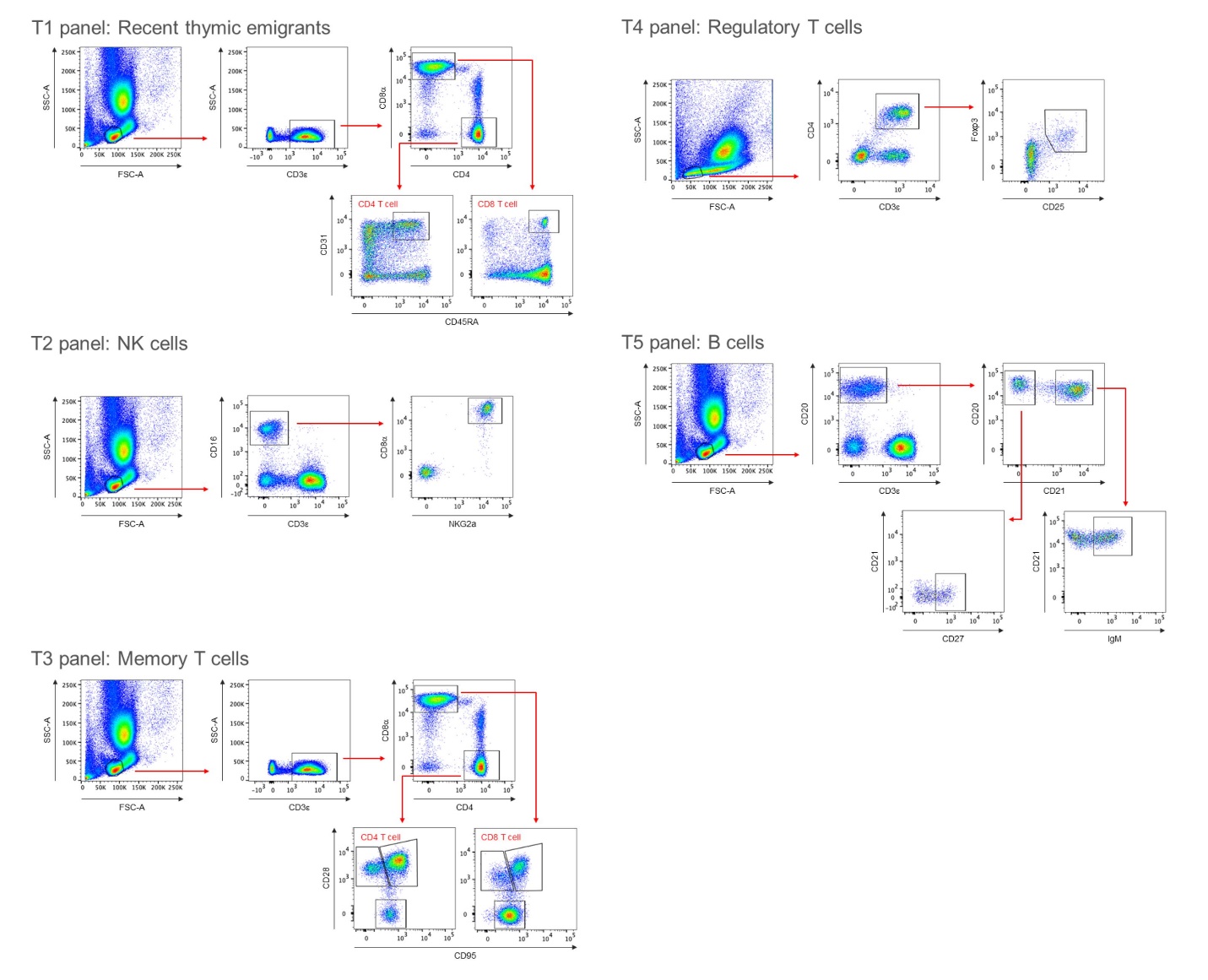


**Supplementary Figure 4.** **Gating strategy for lymphocyte analysis.** Lymphocyte subsets from peripheral blood were analyzed monitored throughout the experiment. Lymphocytes were first gated based on size and granularity, cells selected for CD3ε positive staining and further for CD4+ and CD8+ T cells. (a) Gating was then applied to select recent thymic emigrants (CD31+, CD45RA+). (b) Naïve (CD95-CD28+), central memory (CD95+CD28+), and effector memory (CD95+CD28-) T cells. (c) Regulatory T cells were gated by selecting CD4+ cells and further gating for CD25+Foxp3+ cells. (d) NK cells were gated as (CD3ε-CD16+CD8α+NKG2A+). (e) B cells were selected by CD20 positive staining and further gating applied to distinguish memory B cells (CD21-CD27+) and naïve B cells (CD21+IgM+).


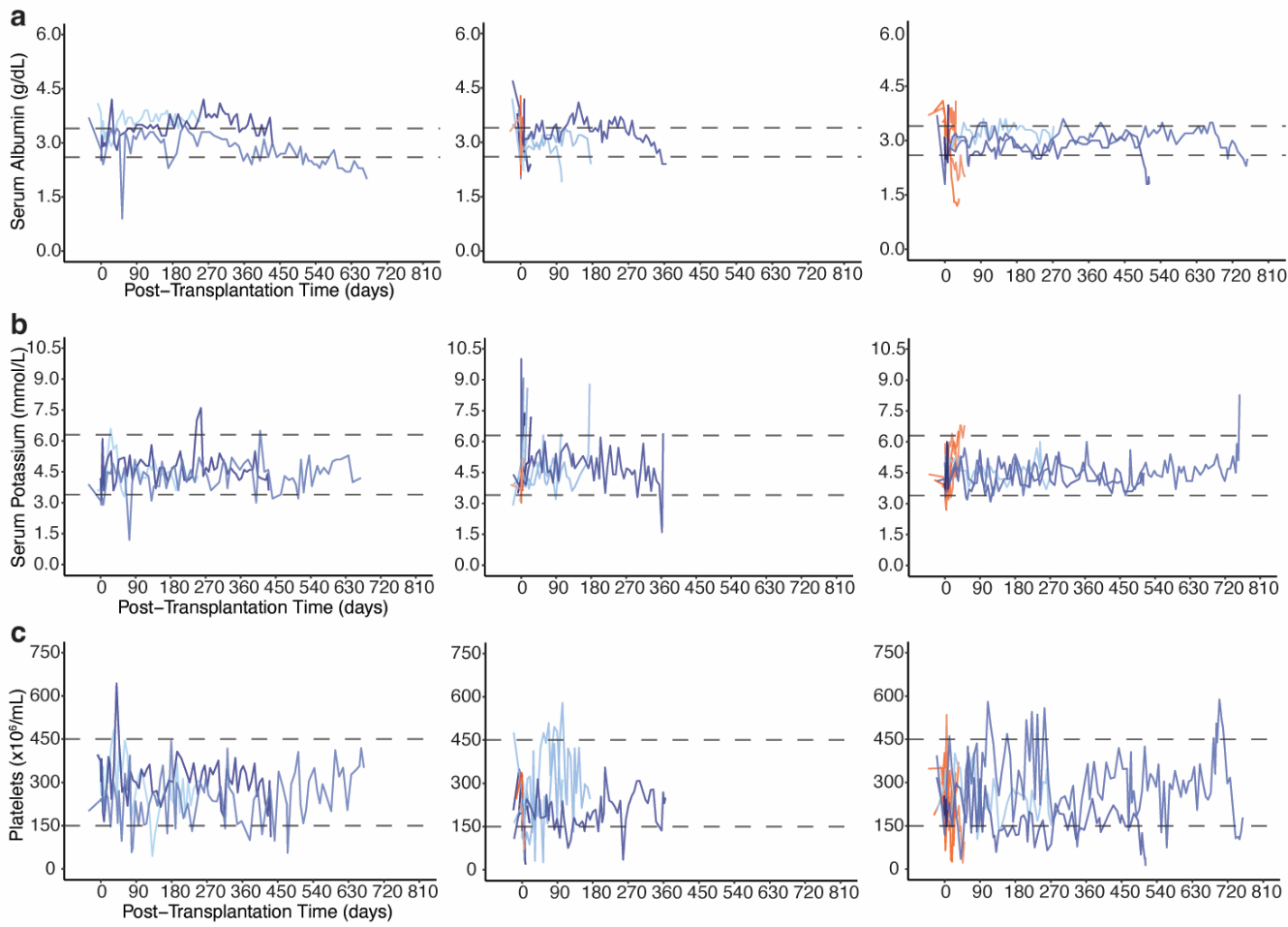


**Supplementary Figure 5**. Additional biomarkers of renal transplantation. **a**. Serum albumin levels generally remained within normal range, except when associated with graft failure, for recipients carrying 3KO.7TG and 3KO.7TG.RI renal grafts. Albumin levels in M11521, a 3KO renal graft recipient, were below normal range throughout transplant (orange line). **b**. Serum potassium levels remained within normal range, except when associated with graft failure, for recipients carrying 3KO.7TG, 3KO.7TG.RI and 3KO ±RI renal grafts. **c**. Platelet counts generally remained within normal range. First column: transplants ongoing at time of manuscript preparation; second column: transplants experiencing renal failure; third column: NHPs euthanized at humane endpoint.

**References**

51. Li, H. *et al.* The Sequence Alignment/Map format and SAMtools. *Bioinformatics* **25**, 2078 (2009).

52. Jain, C., Rhie, A., Hansen, N. F., Koren, S. & Phillippy, A. M. Long-read mapping to repetitive reference sequences using Winnowmap2. *Nat Methods* **19**, 705–710 (2022).

53. Robinson, J. T. *et al.* Integrative Genomics Viewer. *Nat Biotechnol* **29**, 24–26 (2011).

54. Li, H. Minimap2: pairwise alignment for nucleotide sequences. *Bioinformatics* **34**, 3094–3100 (2018).

55. Ewels, P. A. *et al.* The nf-core framework for community-curated bioinformatics pipelines. *Nat Biotechnol* **38**, 276–278 (2020).

56. Pertea, G. & Pertea, M. GFF Utilities: GffRead and GffCompare. *F1000Res* **9**, ISCB Comm J-304 (2020).

57. Patro, R., Duggal, G., Love, M. I., Irizarry, R. A. & Kingsford, C. Salmon provides fast and bias-aware quantification of transcript expression. *Nat Methods* **14**, 417–419 (2017).

58. Soneson, C., Love, M. I. & Robinson, M. D. Differential analyses for RNA-seq: transcript-level estimates improve gene-level inferences. *F1000Res* **4**, 1521 (2015).

59. Love, M. I., Huber, W. & Anders, S. Moderated estimation of fold change and dispersion for RNA-seq data with DESeq2. *Genome Biol* **15**, 550 (2014).

60. Gu, Z., Eils, R. & Schlesner, M. Complex heatmaps reveal patterns and correlations in multidimensional genomic data. *Bioinformatics* **32**, 2847–2849 (2016).

61. Kaminow, B., Yunusov, D. & Dobin, A. *STARsolo: accurate, fast and versatile mapping/quantification of single-cell and single-nucleus RNA-seq data*. http://biorxiv.org/lookup/doi/10.1101/2021.05.05.442755 (2021) doi:10.1101/2021.05.05.442755.

62. Lun, A. T. L. *et al.* EmptyDrops: distinguishing cells from empty droplets in droplet-based single-cell RNA sequencing data. *Genome Biol* **20**, 63 (2019).

63. Germain, P.-L., Lun, A., Garcia Meixide, C., Macnair, W. & Robinson, M. D. Doublet identification in single-cell sequencing data using scDblFinder. *F1000Res* **10**, 979 (2021).

64. Amezquita, R. A. *et al.* Orchestrating single-cell analysis with Bioconductor. *Nat Methods* **17**, 137–145 (2020).

65. Hao, Y. *et al.* Integrated analysis of multimodal single-cell data. *Cell* **184**, 3573-3587.e29 (2021).

66. Lopez, R., Regier, J., Cole, M. B., Jordan, M. I. & Yosef, N. Deep generative modeling for single-cell transcriptomics. *Nat Methods* **15**, 1053–1058 (2018).

67. McCarthy, D. J., Campbell, K. R., Lun, A. T. L. & Wills, Q. F. Scater: pre-processing, quality control, normalization and visualization of single-cell RNA-seq data in R. *Bioinformatics* **33**, 1179–1186 (2017).

68. Balzer, M. S., Rohacs, T. & Susztak, K. How Many Cell Types Are in the Kidney and What Do They Do? *Annu Rev Physiol* **84**, 507–531 (2022).

69. Liberzon, A. *et al.* Molecular signatures database (MSigDB) 3.0. *Bioinformatics* **27**, 1739–1740 (2011).

70. Korotkevich, G. *et al.* *Fast gene set enrichment analysis*. http://biorxiv.org/lookup/doi/10.1101/060012 (2016) doi:10.1101/060012.

71. Clement, K. *et al.* Accurate and rapid analysis of genome editing data from nucleases and base editors with CRISPResso2. *Nat Biotechnol* **37**, 224–226 (2019).

72. Wang, L., Cooper, D. K. C., Burdorf, L., Wang, Y. & Iwase, H. Overcoming Coagulation Dysregulation in Pig Solid Organ Transplantation in Nonhuman Primates: Recent Progress. *Transplantation* **102**, 1050–1058 (2018).

73. Medof, M. E., Kinoshita, T. & Nussenzweig, V. Inhibition of complement activation on the surface of cells after incorporation of decay-accelerating factor (DAF) into their membranes. *J Exp Med* **160**, 1558–1578 (1984).

74. Masaki, T., Matsumoto, M., Nakanishi, I., Yasuda, R. & Seya, T. Factor I-dependent inactivation of human complement C4b of the classical pathway by C3b/C4b receptor (CR1, CD35) and membrane cofactor protein (MCP, CD46). *J Biochem* **111**, 573–578 (1992).

75. Haas, M. *et al.* C4d and C3d staining in biopsies of ABO- and HLA-incompatible renal allografts: correlation with histologic findings. *Am J Transplant* **6**, 1829–1840 (2006).

76. Rosales, I. A. *et al.* Banff Human Organ Transplant Transcripts Correlate with Renal Allograft Pathology and Outcome: Importance of Capillaritis and Subpathologic Rejection. *J Am Soc Nephrol* **33**, 2306–2319 (2022).
