# Supplementary material for: Design and testing of a humanized porcine donor for xenotransplantation
Source: Nature. 2023 Oct 11;622(7982):393–401. doi: 10.1038/s41586-023-06594-4 (PMC10567564; doi:10.1038/s41586-023-06594-4)
Supplement: Supplementary file 2 — Reporting Summary [file 41586_2023_6594_MOESM2_ESM.pdf]

nature portfolio

Corresponding author(s): Wenning Qin, Michele Youd

Last updated by author(s): Aug 3, 2023

Reporting Summary

Nature Portfolio wishes to improve the reproducibility of the work that we publish. This form provides structure for consistency and transparency in reporting. For further information on Nature Portfolio policies, see our Editorial Policies and the Editorial Policy Checklist.

Statistics

For all statistical analyses, confirm that the following items are present in the figure legend, table legend, main text, or Methods section.

n/a	Confirmed	
☐	☒	The exact sample size (*n*) for each experimental group/condition, given as a discrete number and unit of measurement
☐	☒	A statement on whether measurements were taken from distinct samples or whether the same sample was measured repeatedly
☐	☒	The statistical test(s) used AND whether they are one- or two-sided<br>*Only common tests should be described solely by name; describe more complex techniques in the Methods section.*
☒	☐	A description of all covariates tested
☐	☒	A description of any assumptions or corrections, such as tests of normality and adjustment for multiple comparisons
☐	☒	A full description of the statistical parameters including central tendency (e.g. means) or other basic estimates (e.g. regression coefficient) AND variation (e.g. standard deviation) or associated estimates of uncertainty (e.g. confidence intervals)
☐	☒	For null hypothesis testing, the test statistic (e.g. *F*, *t*, *r*) with confidence intervals, effect sizes, degrees of freedom and *P* value noted<br>*Give P values as exact values whenever suitable.*
☒	☐	For Bayesian analysis, information on the choice of priors and Markov chain Monte Carlo settings
☒	☐	For hierarchical and complex designs, identification of the appropriate level for tests and full reporting of outcomes
☐	☒	Estimates of effect sizes (e.g. Cohen's *d*, Pearson's *r*), indicating how they were calculated

Our web collection on statistics for biologists contains articles on many of the points above.

Software and code

Policy information about availability of computer code

Data collection	BD FACSymphony A3 cytometerr; Zeiss AxioScan.Z1 automated whole slide fluorescence scanner; Envision 2105 Multimode Plate reader; SpectraMax FilterMax F5 Plate reader; Incucyte SX5 Live-Cell Analysis reader
Data analysis	Graphpad Prism v8.2.0; FlowJo v10.6.1; Phoenix WinNonlin, version 8.1 software; IGV version 2.12.3; R v4.1.2; ComplexHeatmap v2.10.0; Incucyte; IncuCyte software v2021A; winnowmap2 v2.03; samtools v1.7; R library DECIPHER; minimap2 v2.22-r1101; gffread v0.12.7; salmon index v1.6.0; tximport v1.22.0; DESeq2 v1.34.0; ggplot2; ComplexHeatmap v2.10.0; STARsolo v2.7.9a; DropletUtils v1.14.2; scDblFinder v1.8.0; scran quickCluster, computeSumFactors, and logNormCounts per scran v1.22.1; Seurat v4.1.0; scVI v0.15.5; scater v1.22.0; scran clusterCells; scran scoreMarkers; fgsea v1.20.0; scuttle v1.6.2; ggplot2 v3.2.0; Zeiss Zen Blue 3.0<br>Code for proper Payload Visualization: https://github.com/egenesis/nanopore_igv_issue_fix_sy.git<br>Code for scRNAseq and RNAseq analysis: https://github.com/egenesis/Nature_2023_RNA-seq; https://github.com/egenesis/Nature_2023_scRNAseq

For manuscripts utilizing custom algorithms or software that are central to the research but not yet described in published literature, software must be made available to editors and reviewers. We strongly encourage code deposition in a community repository (e.g. GitHub). See the Nature Portfolio guidelines for submitting code & software for further information.

Data

Policy information about availability of data

All manuscripts must include a data availability statement. This statement should provide the following information, where applicable:

- Accession codes, unique identifiers, or web links for publicly available datasets
- A description of any restrictions on data availability
- For clinical datasets or third party data, please ensure that the statement adheres to our policy

NCBI Gene Expression Omnibus (GEO; https://www.ncbi.nlm.nih.gov/geo/) and Sequence Read Archive (SRA; https://www.ncbi.nlm.nih.gov/sra) under BioProject PRJNA870308

Research involving human participants, their data, or biological material

Policy information about studies with human participants or human data. See also policy information about sex, gender (identity/presentation), and sexual orientation and race, ethnicity and racism.

Reporting on sex and gender	*Use the terms sex (biological attribute) and gender (shaped by social and cultural circumstances) carefully in order to avoid confusing both terms. Indicate if findings apply to only one sex or gender; describe whether sex and gender were considered in study design; whether sex and/or gender was determined based on self-reporting or assigned and methods used. Provide in the source data disaggregated sex and gender data, where this information has been collected, and if consent has been obtained for sharing of individual-level data; provide overall numbers in this Reporting Summary. Please state if this information has not been collected. Report sex- and gender-based analyses where performed, justify reasons for lack of sex- and gender-based analysis.*
Reporting on race, ethnicity, or other socially relevant groupings	*Please specify the socially constructed or socially relevant categorization variable(s) used in your manuscript and explain why they were used. Please note that such variables should not be used as proxies for other socially constructed/relevant variables (for example, race or ethnicity should not be used as a proxy for socioeconomic status). Provide clear definitions of the relevant terms used, how they were provided (by the participants/respondents, the researchers, or third parties), and the method(s) used to classify people into the different categories (e.g. self-report, census or administrative data, social media data, etc.) Please provide details about how you controlled for confounding variables in your analyses.*
Population characteristics	*Describe the covariate-relevant population characteristics of the human research participants (e.g. age, genotypic information, past and current diagnosis and treatment categories). If you filled out the behavioural & social sciences study design questions and have nothing to add here, write "See above."*
Recruitment	*Describe how participants were recruited. Outline any potential self-selection bias or other biases that may be present and how these are likely to impact results.*
Ethics oversight	*Identify the organization(s) that approved the study protocol.*

Note that full information on the approval of the study protocol must also be provided in the manuscript.

Field-specific reporting

Please select the one below that is the best fit for your research. If you are not sure, read the appropriate sections before making your selection.

☒ Life sciences ☐ Behavioural & social sciences ☐ Ecological, evolutionary & environmental sciences

For a reference copy of the document with all sections, see nature.com/documents/nr-reporting-summary-flat.pdf

Life sciences study design

All studies must disclose on these points even when the disclosure is negative.

Sample size	We did not run an a priori power calculation. Essentially, this was not done because, as the reviewer noted, NHP studies are expensive and complex and we simply do not expect to be able to perform a statistically powered study in NHP. Sample sizes among the groups in our study were chosen based on historical studies from the xenotransplantation community and what is practical.
Data exclusions	Three transplants were excluded from the 3KO.7TG group. These porcine donors were cloned from nuclear donor cells of the EG114-124 edited clonal population of cells, which by NGS analysis was later found to carry a rearranged Payload 15S sequence at the AAVS1 genomic safe harbor site. Due to this complication, expression of the ssEEF1A cassette carrying the TNFAIP3 and HMOX1 genes was compromised. In consultation with Dr. George Caputa (on April 17, 2023), our editor from Nature, we made the decision not to include these 3 transplants for the 3KO.7TG group.
Replication	NHP transplantations were performed over the course of 2 years, from October, 2020, to July 2022, involving 3 academic groups (MGH, Duke, and UW Madison). The survival benefit of the 3KO.7TG+/-RI genotype is consistently achieved, as compared with the 3KO+/-RI group. For in

vitro analyses of the function of the genetic edits on endothelial cells, experiments were performed independently at least twice with similar outcomes.

Randomization

The porcine donors were cloned from two clonal populations of edited cells, EG114-94 or EG114-137, and originated from the same wild type pig, Yuc25F. These two clones were confirmed to carry the 3KO.7TG+/-RI edits by extensive NGS analysis and therefore, used interchangeably. NHP recipients were chosen based on low performed antibody binding to porcine cells, in no particular order. Their assignment into one group over the other is presented in Extended Data Figure 6. The 3KO.7TG porcine donors were the first to come off our production line and their kidneys were transplanted into the NHPs in between 2020 and 2021. Next off the production line were the 3KO.7TG.RI donors, whose kidneys were transplanted between 2021 and 2022. The 3KO+/- RI genotypes were transplanted in 2022. For in vitro studies to investigate the function of the genetic edits on primary endothelial cells, endothelial cells used in these experiments from multiple porcine donors were chosen randomly based on the number of frozen vials we had in our possession. Once we decided on the donors to use,. all the in vitro functional analysis with KECs was performed with cells from the same donors across the different assays. For the studies with AECs, AECs were chosen based on what we had stored in liquid nitrogen.

Blinding

The 3 academic centers (MGH, Duke, and UW Madison) performed the NHP transplantation studies and were not blinded about the genotypes of the porcine donors. We did not believe it was necessary, as it was obvious that the 3KO+/-RI group uniformly did not survive well. For in vitro studies, samples used in the functional analyses were not blinded as we knew the genotypes of the porcine donor cells used in the assays and we knew which NHP recipient (and their porcine donor identity) serum/blood samples were being analyzed.

Reporting for specific materials, systems and methods

We require information from authors about some types of materials, experimental systems and methods used in many studies. Here, indicate whether each material, system or method listed is relevant to your study. If you are not sure if a list item applies to your research, read the appropriate section before selecting a response.

Materials & experimental systems

n/a	Involved in the study
☐	☒ Antibodies
☐	☒ Eukaryotic cell lines
☒	☐ Palaeontology and archaeology
☐	☒ Animals and other organisms
☒	☐ Clinical data
☒	☐ Dual use research of concern
☒	☐ Plants

Methods

n/a	Involved in the study
☒	☐ ChIP-seq
☐	☒ Flow cytometry
☒	☐ MRI-based neuroimaging

Antibodies

Antibodies used

Antibody/Reagent Clone Vendor Catalogue Number
Unlabeled anti-EPCR/CD201 clone OTI12H5 abcam ab236517
Unlabeled anti-TM clone PBS-01 abcam ab6980
Unlabeled anti-A20 clone 59A426 Thermo Fisher MA5-16164
Unlabeled anti-HO-1 clone EP1391Y abcam ab52947
Unlabeled anti-CD46 clone EPR4014 abcam ab108307
Unlabeled anti-CD55 clone EPR6689 abcam ab133684
Unlabeled anti-CD47 clone SP279 abcam ab226837
Goat-anti-mouse-HRP DAKO K4001
Goat-anti-rabbit-HRP DAKO K4003
Isolectin B4-FITC Enzo ALX-650-001F-MC05
DBA-biotin Vector Labs B-1035
Unlabeled chicken anti-Neu5Gc clone Poly21469 BioLegend 146903
Alexa Fluor 647 conjugated goat anti-Chicken Thermo Fisher A21449
Alexa Fluor 568 conjugated Streptavidin Thermo Fisher S11226
Alexa Fluor 488 conjugated F(ab')2 anti-human IgG Jackson ImmunoResearch 109-546-098
Alexa Fluor 647 conjugated F(ab')2 anti-human IgM Jackson ImmunoResearch 109-606-129
PE conjugated anti-C3b clone 3E7/C3b BioLegend 846104
APC/Cy7 conjugated anti-human CD46 clone TRA-2-10 BioLegend 352409
PE conjugated anti-human CD55 clone IA10 BD Biosciences 555694
FITC conjugated anti-human CD47 clone B6H12 Thermo Fisher 11-0479-41
BV650 anti-human TM/CD141 clone 1A4 BD Biosciences 740604
PE conjugated anti-human EPCR/CD201 clone RCR-252 BD Biosciences 557950
Alexa 647 anti-porcine CD31 clone 377537 R&D Systems FAB33871R
Ghost Dye Red 780 viability dye Tonbo Biosciences 13-0865
Unlabeled human CD47-Fc R&D Systems 4670-CD-050
PE conjugated anti-human IgG R&D Systems 409304
PE-Cy7 conjugated anti-human SIRPa clone SE5A5 BioLegend 323807
Unlabeled anti-A20 clone EPR2663 abcam ab92324
IRDye® 800CW conjugated goat anti-rabbit IgG H&L abcam ab216773
Unlabeled anti-actin clone C4/actin BD Biosciences 612656
RDye® 680RD conjugated goat anti-mouse IgG H&L abcam ab216776

anti-CD154 clone 5C8H1D MassBiologics PR-1547
anti-CD154 supplied by Tonix Pharmaceuticals
Polyclonal rabbit anti-rhesus thymocyte globulin MassBiologics PR-10027
Anti-CD20-Afucosylatedclone clone PR-8288 MassBiologics 2B8R1F8

Validation

These antibodies have been developed to recognize the human orthologous proteins (CD46, CD55, CD47, TM, EPCR, A20, and HO-1). We performed flow cytometry and IHC experiments to make sure that they do not cross react with the cognate porcine proteins by including control porcine cells that did not express the human orthollouge:
Unlabeled anti-EPCR abcam ab236517
Unlabeled anti-TM abcam ab6980
Unlabeled anti-A20 ThermoFisher MA5-16164
Unlabeled anti-HO-1 abcam ab52947
Unlabeled anti-CD46 abcam ab108307
Unlabeled anti-CD55 abcam ab133684
Unlabeled anti-CD47 abcam ab226837
APC/Cy7 conjugated anti-human CD46 BioLegend 352409
PE conjugated anti-human CD55 BD Biosciences 555694
FITC conjugated anti-human CD47 Invitrogen 11-0479-42
BV650 conjugated anti-PE human thrombomodulin (CD141) BD Biosciences 740604
PE conjugated anti-human endothelial protein C receptor BD Biosciences 557950

These 3 reagents were used to detect the glycan xenoantigens of alpha-Gal, Neu5Gc, and Sd(a). They were validated by positive staining from porcine WT cells and tissues and absence of signal in knockout cells and tissues:
Isolectin B4-FITC Enzo ALX-650-001F-MC05
DBA-biotin Vector Labs B-1035
Unlabeled chicken anti-Neu5GC BioLegend 146903

This reagent was validated to cross react with both the human and cyno C3b proteins by flow cytometry studies:
PE conjugated anti-human C3b/iC3b BioLegend 846104

This reagent was validated to react with porcine CD31 but not human CD31 by IHC & flow cytometry experiment:
Alexa647 anti-porcine CD31 R&D Systems FAB33871R

This antibody was developed to human A20 and validated on cells expressing human A20 compared to control cells not expressing human A20 by Western blot:
Unlabeled anti-A20 clone EPR2663 abcam ab92324

These two antibodies were developed from the original 5c8 clone that was first described in 1992 (S. Lederman et al. 1992 J. Exp. 175: 1091-1101). The original humanized 5c8 clone was in clinical development in the late 90s/early 2000s, has been extensively studied and shown to specifically bind to human, and cross-react to NHP, CD40L. Both of these antibodies have the same 5c8 dervied VDJ antigen binding region but differ in their Fc domain. Given this, we felt comfortable that these two 5c8 versions used here were specific for the CD40L antigen and cross-reactive to cyno CD40L, and we did not perform additional validation studies:
anti-CD154 clone 5C8H1D MassBiologics PR-1547
anti-CD154 supplied by Tonix Pharmaceuticals

These two antibodies have been validated by the vendor to bind to NHP T cells (for anti-rhesus thymocyte globulin) and B cells (for anti-CD20) and both deplete these immune cell subsets. We did not perform independent in vitro validation studies but our analysis of circulating immune cell subsets in the NHP recipients shows T cell and B cell depletion, indicates that both antibodies bind to and deplete their intended targets and cells.
Polyclonal rabbit anti-rhesus thymocyte globulin MassBiologics PR-10027
Anti-CD20-Afucosylatedclone clone PR-8288 MassBiologics 2B8R1F8

These reagents were validated on human and cyno monocytes expressing SIRPa by flow cytometry:
Unlabeled human CD47-Fc R&D Systems 4670-CD-050
PE-Cy7 conjugated anti-human SIRPa clone SE5A5 BioLegend 323807

This antibody is known to react to all actin isoforms in vertebrae muscle and non-muscle cells:
Unlabeled anti-actin clone C4/actin BD Biosciences 612656

All secondary antibodies were validated by showing the lack of staining when the primary antibody was absent by IHC & flow cytometry.

Eukaryotic cell lines

Policy information about cell lines and Sex and Gender in Research

Cell line source(s)

Primary porcine cells were derived by eGenesis from fresh tissues (ear punch biopsy, aorta, and kidney). HUVECs were purchased from ATCC (catalog #PCS-100-010). HMGVECs were purchased from Cell Systems (catalog # ACBRI-128). Human CD47 expressing Jurkat cells and untransfected Jurkats were putchased from Eurofins (93-1135Y19).

Authentication

NGS was performed to extensively analyze the cells isolated from pigs and genotypes of each porcine donor confirmed.

Authentication	HUVECs, HMGVECs, and Jurkat cells were authenticated by flow cytometry analysis of human protein expression and CD47 expression for the Jurkat cells.
Mycoplasma contamination	Primary porcine cells were not routinely tested for mycoplasma. Commercial cell lines were tested and reported as negative.
Commonly misidentified lines (See ICLAC register)	We do not believe any lines used in our study are among these.

Animals and other research organisms

Policy information about studies involving animals; ARRIVE guidelines recommended for reporting animal research, and Sex and Gender in Research

Laboratory animals	We used two animal species in our study. The Yucatan miniature pig, Yuc25F, was female and her ear punch biopsy procured at birth from PremierBiosciences and cells derived from the ear punch biopsy sample genetically modified by eGenesis. The porcine donors used in the transplant studies were approximately 8 weeks of age. Cynomolgus macaques, males and females, were purchased from Bioculture US LLC and Alpha Genesis, weighing 4-12 kg and with an estimated age of 3-8 years old.
Wild animals	We do not use these in our study.
Reporting on sex	The porcine donors were female. The cyno recipients were both males and females.
Field-collected samples	No field collected studies were used in this study.
Ethics oversight	For NHP studies, all animal care, surgical procedures, and postoperative care of animals were conducted in accordance with National Institutes of Health Guidelines for the care and use of primates and the Guide for the Care and Use of Laboratory Animals and were approved by IACUCs at Duke University (Protocol A032-20-02, approved 02/27/2020), University of Wisconsin at Madison (Protocol G006507, approved 9/30/2021), and the Massachusetts General Hospital (Protocol 2017N000216, approved 11/20/2020). Animal cloning was performed under Institutional Animal Care and Use Committee (IACUC)-approved protocols (eGenesis Wisconsin Protocol HF2020-01, approved 11/24/2020, and Precigen Exemplar Protocol MRP2018-003, approved 6/21/2018). All donor production strictly followed the Guide for the Care and Use of Laboratory Animals (National Research Council of National Academies). All animal studies adhered to the 3R principles.

Note that full information on the approval of the study protocol must also be provided in the manuscript.

Flow Cytometry

Plots

Confirm that:

☒ The axis labels state the marker and fluorochrome used (e.g. CD4-FITC).

☒ The axis scales are clearly visible. Include numbers along axes only for bottom left plot of group (a 'group' is an analysis of identical markers).

☒ All plots are contour plots with outliers or pseudocolor plots.

☒ A numerical value for number of cells or percentage (with statistics) is provided.

Methodology

Sample preparation	For lymphocyte depletion studies: peripheral blood was drawn, peripheral blood mononuclear cells were isolated by density gradient centrifugation with lymphoprep (STEMCELL Technologies, 07801) SepMate™-50 (IVD) tubes (STEMCELL Technologies, 85450) according to the manufacturer’s instructions and stained.<br><br>For other studies: Primary porcine cells were harvested via trypLE treatment, washed, and stained with appropriate antibodies, washed and acquired or sorted as described in our Materials and Methods section.
Instrument	FACS Symphony A3 cyto; Beckman Coulter MoFlo Astrios EQ, BD FACSMelody, or Thermo Fisher Bigfoot Spectral cell sorter
Software	FlowJo software
Cell population abundance	Over 95% pure based on cell-specific marker expression (CD31 or transgene) post-sort.
Gating strategy	For T and B cell depletion from immune suppression regimen:<br>First gate: VSC versus SSC for scatter of cells<br>Second gate: SSC versus CD3 to select for T cells<br>Third gate: Pending on cell type intended to be selected, this differs.<br>Gating strategy is provided in Supplementary Information Figure 3.<br><br>For all other studies:<br>First gate: FSC versus SSC for scatter of cells<br>Second gate: FSC-A versus FSC-H to select for singlets<br>Third gate: Live/dead staining to select live cells

Fourth gate: CD31 staining to select for ECs
Gating strategy is provided in Supplementary Information Figure 2.

☒ Tick this box to confirm that a figure exemplifying the gating strategy is provided in the Supplementary Information.
